# R1441G but not G2019S mutation enhances LRRK2 mediated Rab10 phosphorylation in human peripheral blood neutrophils

**DOI:** 10.1007/s00401-021-02325-z

**Published:** 2021-06-14

**Authors:** Ying Fan, Raja S. Nirujogi, Alicia Garrido, Javier Ruiz-Martínez, Alberto Bergareche-Yarza, Elisabet Mondragón-Rezola, Ana Vinagre-Aragón, Ioana Croitoru, Ana Gorostidi Pagola, Laura Paternain Markinez, Roy Alcalay, Richard A. Hickman, Jonas Düring, Sara Gomes, Neringa Pratuseviciute, Shalini Padmanabhan, Francesc Valldeoriola, Leticia Pérez Sisqués, Cristina Malagelada, Teresa Ximelis, Laura Molina Porcel, Maria José Martí, Eduardo Tolosa, Dario R. Alessi, Esther M. Sammler

**Affiliations:** 1 Medical Research Council Protein Phosphorylation and Ubiquitylation Unit, University of Dundee, Dundee, DD1 5EH UK; 2 Parkinson’s Disease and Movement Disorders Unit, Neurology Service, Hospital Clínic de Barcelona, Barcelona, Spain; 3 Centro de Investigación Biomédica en Red Sobre Enfermedades Neurodegenerativas (CIBERNED), Hospital Clínic, IDIBAPS, Universitat de Barcelona, Barcelona, Spain; 4 Group of Neurodegenerative Diseases, Biodonostia Research Institute, San Sebastian, Spain; 5 Department of Neurology, Columbia University Medical Center, New York, NY USA; 6 Department of Pathology and Cell Biology, Columbia University Medical Center, New York, NY USA; 7 The Michael J Fox Foundation for Parkinson’s Research, New York, NY USA; 8 Departament de Biomedicina, Facultat de Medicina I Ciències de La Salut, Institut de Neurociències, Universitat de Barcelona, Barcelona, Catalonia Spain; 9 Centro de Investigación Biomédica en Red Sobre Enfermedades Neurodegenerativas (CIBERNED), Madrid, Spain; 10 Neurological Tissue Bank of the Biobanc-Hospital Clinic-Institut D’Investigacions Biomediques August Pi I Sunyer (IDIBAPS), Barcelona, Spain; 11 Alzheimer’s Disease and Other Cognitive Disorders Unit, Neurology Service, Hospital Clínic, Institut D’Investigacions Biomediques August Pi I Sunyer (IDIBAPS), University of Barcelona, Barcelona, Spain; 12 Molecular and Clinical Medicine, Ninewells Hospital and Medical School, University of Dundee, Dundee, DD1 9SY UK

**Keywords:** Parkinson’s disease, LRRK2, LRRK2 kinase inhibitors, RabGTPases, Biomarkers, Protein phosphorylation

## Abstract

Heterozygous gain-of-kinase function variants in *LRRK2* (leucine-rich repeat kinase 2) cause 1–2% of all cases of Parkinson’s disease (PD) albeit with incomplete and age-dependent penetrance. All pathogenic LRRK2 mutations reside within the two catalytic domains of LRRK2—either in its kinase domain (e.g. G2019S) with modest effect or its ROC-COR GTPase domain (e.g. R1441G/H) with large effect on LRRK2 kinase activity. We have previously reported assays to interrogate LRRK2 kinase pathway activity in human bio-samples measuring phosphorylation of its endogenous substrate Rab10, that mirrors LRRK2 kinase activation status. Here, we isolated neutrophils from fresh peripheral blood from 101 participants including 42 LRRK2 mutation carriers (21 with the G2019S and 21 with the R1441G mutations), 27 patients with idiopathic PD, and 32 controls. Using a dual approach, LRRK2 dependent Rab10 phosphorylation at Threonine 73 (pRab10^Thr73^) was measured by quantitative multiplexed immunoblotting for pRab10^Thr73^/total Rab10 as well as targeted mass-spectrometry for absolute pRab10^Thr73^ occupancy. We found a significant over fourfold increase in pRab10^Thr73^ phosphorylation in carriers of the LRRK2 R1441G mutation irrespective of clinical disease status. The effect of the LRRK2 G2019S mutation did not reach statistical significance. Furthermore, we show that LRRK2 phosphorylation at Serine 935 is not a marker for LRRK2 kinase activity in human neutrophils. When analysing pRab10^Thr73^ phosphorylation in post-mortem brain samples, we observed overall high variability irrespective of clinical and LRRK2 mutation status and attributed this mainly to the adverse effect of the peri- and post-mortem period on the stability of posttranslational modifications such as protein phosphorylation. Overall, in vivo LRRK2 dependent pRab10^Thr73^ phosphorylation in human peripheral blood neutrophils is a specific, robust and promising biomarker for significant LRRK2 kinase hyperactivation, as with the LRRK2 R1441G mutation. Additional readouts and/or assays may be needed to increase sensitivity to detect modest LRRK2 kinase activation, as with the LRRK2 G2019S mutation. Our assays could be useful for patient stratification and target engagement studies for LRRK2 kinase inhibitors.

**Supplementary Information:**

The online version contains supplementary material available at 10.1007/s00401-021-02325-z.

## Introduction

Parkinson’s disease (PD) is a common neurodegenerative condition that affects 1% of people over the age of 60 and over 6 million people worldwide [5]. People with PD present with a wide spectrum of progressive motor and non-motor symptoms reflecting the relentless loss of neurons and neuronal function that often predate clinical symptom onset by decades [20]. The greatest unmet need in PD are disease modifying treatments that slow or stop disease progression which is further highlighted by the projected doubling of PD cases over the next 20 years [5]. While the underlying cause for PD is largely unknown, the discovery of rare genetic forms of the condition has provided crucial insight into pathomechanistic processes altered in PD that have been leveraged for devising novel targeted treatment strategies [19].

The Leucine-rich repeat kinase 2 (LRRK2) is such a highly pursued therapeutic target. It is a large multidomain protein made up of 51 exons and 2537 amino acids with a predicted molecular weight of 286 kDaltons. Its catalytic core is made up of a kinase and ROC-COR GTPase domain as well as other protein–protein interaction motifs: the N-terminal armadillo, ankyrin and leucine-rich repeat motifs and a C-terminal WD40 motif [1]. LRRK2 is highly expressed in immune cells including peripheral blood neutrophils and monocytes as well as lung, kidney and intestine but expression is lower in brain [24, 43]. Pathogenic variants in LRRK2 are a direct cause for PD albeit with age-dependent and incomplete penetrance and cluster within the two catalytic domains of LRRK2 [6]. The substitution of serine for glycine at position 2019 within its kinase domain is the most common PD associated variant and accounts for 1% of sporadic and 4% of familial PD cases in most Caucasian populations worldwide, but up to 29% and 37% of familial cases in Ashkenazi Jews and North African Berbers, respectively, while being largely absent in Asian populations [16, 19, 42]. The much rarer I2020T mutation is also located in the LRRK2 kinase domain. The second most common mutation hotspot is in the ROC-COR GTPase domain including R1441C/G/H/S as well as N1437H and Y1699C [1]. Of these, the R1441G mutation is particularly common in the Basque region in Spain where it is responsible for 46% of all familial PD [26, 42]. In addition to these clearly pathogenic mutations, genome wide association studies have implicated variants at the LRRK2 locus as risk factors for idiopathic PD (iPD) with a modest increase in lifetime susceptibility for PD [30, 31].

All pathogenic LRRK2 mutations have in common that they augment kinase activity suggesting that inhibition of the LRRK2 kinase with small molecules is a promising strategy for disease modification. The LRRK2 kinase domain mutations—G2019S and I2020T—increase LRRK2 kinase activity only modestly, under twofold in in vitro and in vivo cell and animal studies (reviewed in [40]) by domain disruption. The LRRK2 ROC and COR GTPase domain mutations—N1437H, R1441 hotspot and Y1699C—suppress GTPases activity and promote GTP binding which in turn mediates a 3–4-fold increase in LRRK2 kinase activity [38, 39]. Assessing LRRK2 kinase activation status in human bio-samples on the other hand has been a challenge. For example, while LRRK2 autophosphorylation at Serine 1292 correlates with LRRK2 kinase activity [38], its low stoichiometry makes its detection difficult and unreliable. Serine 935 is one of the constitutively phosphorylated LRRK2 sites that reside in a non-catalytic region of LRRK2 and plays a role in 14-3-3 binding [33]. Serine 935 phosphorylation of LRRK2 is one of the principal pharmacodynamic markers for in vivo LRRK2 kinase inhibition [15], but not for LRRK2 kinase activity. More recently, the discovery of a subgroup of RabGTPases, including Rab10, as endogenous LRRK2 kinase substrates that are phosphorylated at a conserved Threonine or Serine in conserved switch II domains [39], the availability of relevant tools [24] and technologies [21] as well as the exploration of novel biomatrices [12] has opened up new opportunities to assess how LRRK2 mutations impinge on is kinase activity.

We have previously described a robust and facile assay for interrogating LRRK2 kinase pathway activity in peripheral blood neutrophils by quantifying LRRK2 mediated phosphorylation of Rab10 at Threonine 73 [11]. Phosphorylation of Rab10 at Threonine 73 (pRab10^Thr73^) is quantified by either immunoblot analysis deploying highly sensitive monoclonal phosphospecific antibodies raised against the pRab10^Thr73^ epitope [11] or by a targeted mass-spectrometry approach [21]. As a biomatrix, peripheral blood neutrophils lend themselves for the study of the LRRK2 pathway as they constitute a homogenous and abundant pool of cells that contain relatively high protein copy numbers of both LRRK2 and Rab10 [11, 12]. We have recently utilized this assay to demonstrate that LRRK2 dependent pRab10^Thr73^ phosphorylation is significantly increased in PD patients carrying a heterozygous VPS35 D620N mutation that augments LRRK2 kinase activity by a yet unknown mechanism. To date elevated LRRK2 kinase pathway activation with LRRK2 dependent pRab10^Thr73^ phosphorylation as a readout has not been demonstrated in bio-samples derived from LRRK2 mutation carriers.

In this study, we have analysed LRRK2 dependent pRab10^Thr73^ phosphorylation as a marker for in vivo LRRK2 activation status in human peripheral blood neutrophils isolated from 101 individuals including 42 LRRK2 mutation carriers (21 with the G2019S mutation that resides in the kinase domain and 21 with the R1441G mutation that lies within the ROC-COR GTPase domain) with and without PD and compared them with 32 healthy controls and 27 individuals with iPD. We show that LRRK2 dependent pRab10^Thr73^ phosphorylation is significantly elevated over fourfold in all R1441G LRRKR2 mutation carriers irrespective of disease status while PD manifesting and non-manifesting G2019S mutation carriers as well as iPD samples lacked any such enhancement over controls. We deployed two independent methodologies—quantitative multiplexed immunoblotting for pRab10^Thr73^ normalized against total Rab10 levels as well as targeted pRab10^Thr73^ occupancy mass-spectrometry and found relatively good correlation between the two assays. Furthermore, we have analysed brain samples derived from eight G2019S LRRK2 and one R1441H LRRK2 mutation carriers as well as ten individuals with iPD and ten controls for LRRK2 dependent pRab10^Thr73^ phosphorylation by immunoblotting. We found high variability amongst donors irrespective of genetic and disease state and concluded that post-mortem brain tissue is unsuitable for analysing posttranslational modifications such as pRab10^Thr73^ phosphorylation.

## Materials and methods

A comprehensive list of reagents, antibodies, cDNA constructs, cell lines, buffers, equipment, software packages utilized in this study are provided in Supplementary Table 1, online resource.

### Reagents

*Cis*-2,6-dimethyl-4-(6-(5-(1-methylcyclopropoxy)-1H-indazol-3-yl)pyrimidin-4-yl)morpholine (MLi-2) [13, 37] was synthesised at the University of Dundee and used at a concentration of 200 nM for a duration of 30 min for LRRK2 kinase inhibition in the peripheral blood neutrophil experiments. Diisopropylfluorophosphate (DIFP) was purchased from Sigma (Cat# D0879), Microcystin-LR was from Enzo Life Sciences and sequencing grade modified trypsin from Promega (Cat# V511A). Complete protease and phosphatase inhibitor tablets were from Roche. All heavy and light stable isotope synthetic peptides described in supplementary Table 1 were synthesized by JPT peptide technologies (https://www.jpt.com/) in 1 nmol aliquots. All synthetic peptides were quantified by amino acid analysis and liquid chromatography–mass-spectrometry (LC–MS) analysis by JPT and confirmed to be of purity of > 95%. The peptides were delivered in a lyophilized form and were resuspended in solvent containing 0.1% (v/v) formic acid in 3% (v/v) acetonitrile to give a final concentration of 10 pmol/ml. Aliquots of this were further diluted in a series of tenfold dilution to a lowest concentration of 10 fmol/ml and stocks of each dilution aliquoted and stored at − 80 °C.

### Antibodies

The recombinant MJFF-pRab10 (Thr73) rabbit monoclonal antibodies were recently described in terms of their high selectivity and specificity [24] and is available from Abcam (Cat# ab230261). The MJFF-total Rab10 mouse antibody was from nanoTools (Cat# 0680–100/Rab10-605B11). Rabbit monoclonal antibodies for total LRRK2 (N-terminus, residues 100–500, UDD3) and phospho-Ser935 LRRK2 (UDD2) as well as the sheep polyclonal antibodies for LRRK1 (sheep number S405C, 2nd bleed) were expressed and purified at the University of Dundee as described previously [7, 10, 25] and are available from MRC PPU Reagents and Services (https://mrcppureagents.dundee.ac.uk/). The sheep polyclonal PPM1H antibodies against the full length PPM1H protein have been described before [2] and are also available from MRC PPU Reagents and Services (sheep number DA018, https://mrcppureagents.dundee.ac.uk/). The C-terminal total LRRK2 mouse monoclonal antibody was from Neuromab (Cat# 75-253). Additionally, we used the rabbit monoclonal MJFF-pRab8 (Thr72) (#ab230260, Abcam, Inc., [24]), mouse monoclonal anti-Rab8A (#WH0004218M2, Sigma–Aldrich), rabbit monoclonal pAMPK (Thr172) (#4188, CST), and mouse monoclonal total AMPK (CST, #2793) antibodies. The mouse anti-GAPDH total was from Santa Cruz Biotechnology (Cat# sc-32233). The rabbit polyclonal pSer72 Rab7A was recently described and was generated by The Michael J. Fox Foundation’s research tools program in partnership with Abcam [25]. Development of a monoclonal antibody is underway. Please contact tools@michaeljfox.org with questions. Recombinant antibodies were used at 1 mg/ml final concentration for immunoblotting except for anti-pRab10 (Thr73) antibody which was used at 0.5 μg/ml final concentration. For immunoblotting applications all commercial monoclonal antibodies were diluted in 5% (w/v) bovine serum albumin in TBS-T (20 mM Tris base, 150 mM Sodium Chloride (NaCl), 0.1% (v/v) Tween20), and sheep polyclonal antibodies were diluted in 5% (w/v) skimmed milk in TBS-T. Goat anti-mouse IRDye 800CW (Cat# 926–32,210) and IRDye 680LT (Cat# 926-68020), goat anti-rabbit IRDye 800CW (Cat# 926-32211) secondary antibodies were from LICOR and used at 1:10,000 dilution in TBS-T.

### Study participants and blood sample collection

Fresh blood was collected from a total of 101 participants; 66 individuals were recruited via the movement disorder clinics at the Hospital Clinic Universitari de Barcelona in the fall of 2017 and 35 individuals via the Hospital Universitario Donostia in San Sebastian in the Basque region of Spain during 2019. Of the 101 participants, 42 carried a pathogenic mutation in LRRK2–21 carried the G2019S mutation that resides in the kinase domain and 21 with the R1441G mutation that lies within the ROC-COR GTPase domain-, 27 patients with iPD, and 32 controls. Demographics such as sex, age, disease duration and age at PD onset were collected. PD diagnosis was defined according to the UK Brain Bank criteria with the exception that a positive family history for PD was not considered an exclusion criteria [17]. Severity of motor symptoms and the presence of motor complications was assessed using part III and IV of the Movement Disorder Society—Unified Parkinson’s disease rating scale (MDS-UPDRS-III, -IV) [14]. Levodopa-equivalent daily dose (LEDD) was recorded as well as any additional non-oral therapies such as Deep Brain Stimulation (DBS). Detailed demographic and clinical information of all participants can be found in Supplementary Table 2, online resource.

### Ethical approval and consent to participate

The study was approved by the respective local ethics committees. All participants gave written informed consent.

### Neutrophil isolation, treatment with the specific LRRK2 kinase inhibitor MLi-2 and cell lysis

Peripheral blood neutrophils were isolated directly from fresh blood using EasySep Direct Human Neutrophil Isolation Kit (Stemcell Technologies, Cat# 19,666) based on an immunonegative magnetic selection process as described before [11, 12]. Pure and alive neutrophils were then pelleted by centrifugation at 335 g for 5 min and resuspended in 20 ml RPMI 1640 media. At this stage, purified neutrophils were divided equally into two tubes for treatment either with the specific LRRK2 kinase inhibitor MLi-2 at a final concentration of 200 nM or with vehicle (DMSO) for 30 min. After MLi-2 treatment, neutrophils were pelleted via centrifugation at 335 g for 5 min and the supernatant was carefully and fully removed before cell lysis in 150 µl of ice-cold lysis buffer [50 mM Tris/HCl, pH 7.5, 1% (v/v) Triton X-100, 1 mM ethylene glycol-bis(β-aminoethyl ether)-*N*,*N*,*N*′,*N*′-tetraacetic acid (EGTA), 1 mM sodium orthovanadate, 50 mM sodium fluoride (NaF), 0.1% (v/v) 2-mercaptoethanol, 10 mM 2-glycerophosphate, 5 mM sodium pyrophosphate, 1 μg/ml mycrocystin-LR (Enzo Life Sciences), 270 mM sucrose, 0.5 mM diisopropylfluorophosphate (DIFP) (Sigma, Cat# D0879) in addition to Complete EDTA-free protease inhibitor cocktail (Roche, Cat# 11836170001)]. Cell lysates were kept on ice for 10 min and then clarified by centrifugation at 20,800*g* for 15 min at 4 °C. Supernatants were used for Bradford assay (Thermo Scientific) and stored at − 80 °C after snap freezing.

### Brain sample preparation

Brain samples were obtained from institutionally approved autopsy collections held by the Columbia University Medical Center in New York, USA and the IDIBAPS Biobank at the Hospital Clinic in Barcelona, Spain.

Frozen human matched frontal (Brodmann area 9) and occipital (Brodmann area 17) cortex samples from nine individuals including three controls, three G2019S mutation carriers with PD and three with iPD were obtained from the brain bank at the Columbia University Medical Center in New York. Additionally, we received 20 frontal cortex samples including seven controls, seven iPD, fife G2019S and one R1441H mutation carriers with PD from the IDIBAPS Biobank at the Hospital Clinic in Barcelona, Spain. Brain samples were weighed and added to a tenfold volume excess of ice-cold lysis buffer [50 mM Tris–HCl pH 7.5, 1% (v/v) Triton X-100, 1 mM EGTA, 1 mM sodium orthovanadate, 50 mM sodium fluoride, 10 mM β-glycerophosphate, 5 mM sodium pyrophosphate, 1 µg/ml microcystin-LR (Enzo Life Sciences), 270 mM sucrose and complete EDTA-free protease inhibitor cocktail (Roche, Cat # 11836170001)], and homogenised using POLYTRON homogenizer (KINEMATICA) on ice (5 s homogenisation, 10 s interval and 5 s homogenisation). Lysates were cleared by centrifugation at 20 800*g* for 10 min at 4 °C. Supernatants were collected, quantified by the Bradford assay (Thermo Scientific) and subjected to immunoblot analysis.

### Quantitative multiplexed immunoblot analysis

Cell lysates were mixed with 4 × SDS–PAGE loading buffer [250 mM Tris–HCl, pH 6.8, 8% (w/v) SDS, 40% (v/v) glycerol, 0.02% (w/v) Bromophenol Blue and 4% (v/v) 2-mercaptoethanol] to a final total protein concentration of 1 µg/µl and heated at 70 °C for 10 min. 10 µg of each sample was loaded onto a NuPAGE 4–12% Bis–Tris Midi Gel (Thermo Fisher Scientific, Cat# WG1403BOX) and electrophoresed at 130 V for 2 h with the NuPAGE MOPS SDS running buffer (Thermo Fisher Scientific, Cat# NP0001-02). At the end of electrophoresis, proteins were electrophoretically transferred onto a nitrocellulose membrane (GE Healthcare, Amersham Protran Supported 0.45 µm NC) at 100 V for 90 min on ice in transfer buffer (48 mM Tris–HCl and 39 mM glycine). Transferred membrane was blocked with 5% (w/v) skim milk powder dissolved in TBS-T [20 mM Tris–HCl, pH 7.5, 150 mM NaCl and 0.1% (v/v) Tween 20] at room temperature for 1 h. The membrane was then cropped into three pieces, namely the ‘top piece’ (from the top of the membrane to 75 kDa), the ‘middle piece’ (between 75 and 30 kDa) and the ‘bottom piece’ (from 30 kDa to the bottom of the membrane). The top piece was incubated with rabbit anti-LRRK2 pS935 UDD2 antibody multiplexed with mouse anti-LRRK2 C-terminus total antibody diluted in 5% (w/v) skim milk powder in TBS-T to a final concentration of 1 µg/ml for each of the antibody. The middle piece was incubated with mouse anti-GAPDH antibody diluted in 5% (w/v) skim milk powder in TBS-T to a final concentration of 50 ng/ml. The bottom piece was incubated with rabbit MJFF-pRAB10 monoclonal antibody multiplexed with mouse MJFF-total Rab10 monoclonal antibody diluted in 2% (w/v) bovine serum albumin in TBS-T to a final concentration of 0.5 µg/ml for each of the antibody. All blots were incubated in primary antibody overnight at 4 °C. Prior to secondary antibody incubation, membranes were washed three times with TBS-T for 10 min each. The top and bottom pieces were incubated with goat anti-mouse IRDye 680LT (#926-68020) secondary antibody multiplexed with goat anti-rabbit IRDye 800CW (#926-32211) secondary antibody diluted in TBS-T (1: 10,000 dilution) for 1 h at room temperature. The middle piece was incubated with goat anti-mouse IRDye 800CW (#926-32210) secondary antibody diluted in TBS-T (1: 10,000 dilution) at room temperature for 1 h. Membranes were washed with TBS-T for three times with a 10 min incubation for each wash. Membranes were scanned using the LICOR Odyssey CLx imaging system.

### Quantification and normalization of the LICOR immunoblot analysis

Quantification of the protein bands was performed on the scanned images using the Odyssey Scan band tool in a blinded manner with regards to genotype and clinical status of the participants by an independent analyst using the Image Studio software. Each sample set including DMSO and MLi-2 treated neutrophil lysates for each participant or each autopsy brain lysate was run in duplicates and two independent replicate immunoblotting experiments were performed and used for quantification. The intensities of the total LRRK2 and total Rab10 protein bands were normalized to that of the housekeeping protein GAPDH (total LRRK2/GAPDH and total Rab10/GAPDH) while the specific posttranslational phosphorylation modifications were quantified against the multiplexed total target protein irrespective of modification (Thr73-pRab10/total Rab10 and Ser935-pLRRK2/total LRRK2). Inter-gel variability was controlled for by normalization against the same control sample on each gel per set of experiments. In total, three sets of Western blot experiments run with samples 1–66 (Barcelona set), samples 67–82 (San Sebastian set 1) and 83–101 (San Sebastian set 2) were performed at different time points. To combine Western blot results for all three sets of neutrophil lysates, results for each set were normalized to the average of its controls and then combined.

### LC–MS/MS sample preparation

#### SDS–PAGE separation and in-gel digestion

The method utilized here was adopted according to the recently published paper by Karayel et al. [21]. Briefly, neutrophil lysates were reduced with 5 mM dithiothreitol at 55 °C for 20 min. Samples were brought to room temperature and treated with 40 mM iodoacetamide for 20 min in the dark. 40 μg of total soluble neutrophil protein extract were separated by SDS–PAGE and the gels were stained with colloidal Coomassie blue (Novex). Protein bands spanning 20–30 kDa region were excised, destained with 40% (v/v) acetonitrile in 40 mM ammonium bicarbonate on a Thermomixer for 20 min at room temperature. Destaining solution was discarded and this step was repeated once, until gel pieces were completely destained. Gel pieces were dehydrated with 100% acetonitrile on a Thermomixer for 15 min at room temperature, followed by complete removal and placing of the tubes on ice. The dried gel pieces were reswollen in 150 μl of 20 mM triethylammonium bicarbonate buffer (TEABC) pH7.5 containing 500 ng of sequencing grade modified trypsin and 0.1% (w/v) sodium deoxycholate. Samples were incubated at 37 °C for 16 h. Peptides were extracted by adding 70 μl of 0.5% acetic acid (v/v) in 80% (v/v) acetonitrile to samples and incubated on a shaker for 15 min at room temperature. The liquid containing the peptide extraction was then transferred into a fresh tube. The extraction step was repeated twice with pooling of the peptide solutions with that of the previous step. Peptide solutions were snap frozen with dry ice and dried in a Speedvac evaporator.

#### SDB-RP (Styrenedivinylbenzene—Reversed Phase) purificatio﻿n

SDB-RP tips were prepared in-house by punching two layers of SDB-RP disks using a 16 gauge syringe needle, which were then pushed into a 250 µl pipette tip. Vacuum dried peptides from the previous step were reconstituted with 100 µl of 1% (v/v) trifluoroacetic acid in isopropanol and directly loaded on SDB-RP tips. Samples were centrifuged at 1500*g* for 10 min at room temperature to allow binding of peptides onto the disks. Flowthrough was collected and reloaded onto stage tips for another round of binding. Subsequently, stage tips were washed twice with 100 µl of 1% (v/v) trifluoroacetic acid in isopropanol, and then with 100 µl of 0.2% (v/v) trifluroacetic acid in 3% (v/v) acetonitrile. Wash steps were undertaken by centrifugation at 2000*g* for 6 min at room temperature and the flowthrough was discarded. Peptides were eluted sequentially with freshly prepared 1.25% (v/v) ammonium hydroxide in 50% (v/v) acetonitrile and 1.25% (v/v) ammonium hydroxide in 80% (v/v) acetonitrile. Purified peptides were snap frozen with dry ice and dried in a Speedvac evaporator.

#### Spike in of heavy-labelled peptides and sample loading on Evotips

Sample loading on Evotips was performed as described previously [2] with minor modifications as described below. Vacuum dried peptides were reconstituted in 80 µl of 0.1% (v/v) formic acid in 3% (v/v) acetonitrile buffer and allowed to dissolve at room temperature for 10 min. To calculate absolute phosphorylated pRab10^Thr73^ occupancy, an equimolar mixture of 25 fmol of heavy stable isotope-labelled (SIL) phosphorylated and non-phosphorylated Rab10 counterpart peptides and 25 fmol of PRTC retention time calibration mix were spiked in. The peptide sample mix was then subjected to sonication in a water bath sonicator (Branson) for 10 min and centrifuged at 17,000*g* at room temperature for 10 min. Evotips (EvoSep, Cat #EV2001) were activated by adding 20 µl of buffer B [100% (v/v) acetonitrile in 0.1% (v/v) formic acid] and centrifuged at 800*g* for 1 min. Tips were immersed into 200 µl of isopropanol and then equilibrated by adding 20 µl of buffer A [0.1% (v/v) formic acid in water] and centrifuging at 800*g* for 1 min. This step was repeated one more time. Peptides were loaded on Evotips using gel loading tips and centrifuged at 700*g* for 5 min. The flowthrough was collected, reapplied and centrifuged. Next, the tips were washed twice with buffer A. 100 µl of buffer A was applied to the Evotips which were then placed on the autosampler tray of EvoSep LC system.

### Parallel reaction monitoring (PRM) measurements

**Fig. 1:**
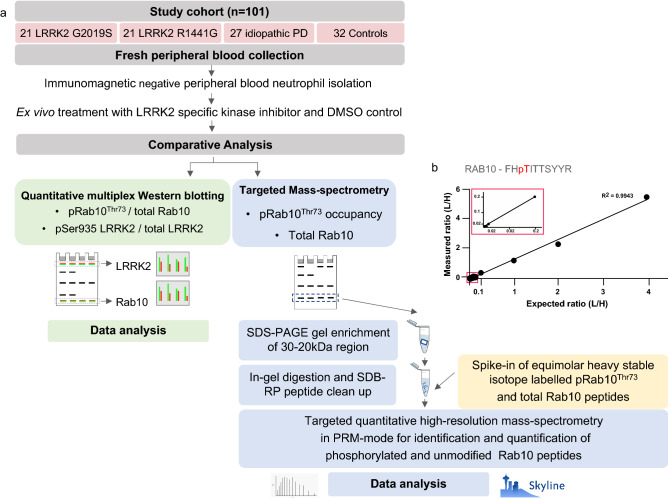
**Experimental design and workflow for peripheral blood neutrophils.a** 20 ml of fresh peripheral blood was collected from 101 participants with and without either PD and with or without a pathogenic LRRK2 mutation—LRRK2 G2019S or LRRK2 R1441G—for peripheral blood neutrophil isolation by immunomagnetic negative isolation. Purified neutrophils were then split into two parts for ex vivo treatment with either the specific LRRK2 kinase inhibitor MLi-2 (200 nM) or DMSO for 30 min prior to cell lysis and storage at -80 degrees Celsius. All sets of neutrophil lysates (MLi-2 and DMSO treated) were then subjected to quantitative multiplexed immunoblotting for pRab10^Thr73^ phosphorylation/total Rab10 protein ratio as well as targeted mass-spectrometry for pRab10^Thr73^ phosphorylation occupancy with spike in of heavy-labelled Rab10-phospho- and total peptide standards after enrichment by SDS–PAGE followed by in-gel digestion and subsequent mass-spectrometry and data analysis. Additionally, quantitative immunoblot analysis was performed for Serine 935 phosphorylation/ total LRRK2 protein as well as total Rab10 and total LRRK2 protein levels, both normalised against GAPDH. **Limit of detection of targeted MS assay** (**b**). Scatter plot depicting the limit of detection and quantification of pRab10^Thr73^ in targeted PRM. 50 fmol of heavy pRab10^Thr73^ was mixed with a variable amount of light pRab10 ranging from 0.01, 0.1, 1, 10, 50, 100 and 200 fmol that was spiked into 50 ng of HeLa lyastes. Light/heavy ratio values were plotted to show the linear response of pRab10^Thr73^ [0.01 to 200 fmol (*R*^2^ = 0.9943)). The zoom in rectangular box depicting the values of 0.01 to 0.1 fmol. *n* = 3, error bars representing mean and SD

All of the targeted LC–MS/MS (PRM) data were acquired on QE HF-X Mass spectrometer interfaced in line with an EvoSep liquid chromatography (LC) system. EvoTips were loaded on EvoSep LC system autosampler tray and analysed using a 21 min (60 samples per day) script. The EvoSep LC system elutes peptides by applying a partial gradient using low pressure pump-A and B into the long storage capillary sample loop where peptides were partially stored and focused by pre-formed gradient using low pressure pump-C and pump-D. Peptides were subsequently directed to an analytical column (Reprosil-pur C18 AQ, 3 µm beads, 100 µm ID, 8 cm long for pRab analysis) by high pressure pump which is housed into the Easy nano source. Peptides were then directly electrosprayed into the mass spectrometer by maintaining 2000 voltage. Mass spectrometer was operated in a targeted PRM mode by employing one full MS scan followed by a PRM scan which is instructed to operate by following the imported inclusion list with a scheduled retention time (peptide sequences and *m*/*z* values are provided in supplementary table 1). Dynamic on-the-fly retention time correction was enabled to correct the scheduled retention time. Full MS scan was acquired within 300–800 *m*/*z* and acquired at 120,000 resolution at 200 *m*/*z* using Orbitrap mass analyser. Each of the targeted analytes was isolated using Quadrupole mass filter with a 0.7 Da isolation window and fragmented using normalized 27% higher energy collisional dissociation (HCD) and measured at 30,000 resolution at 200 *m*/*z* using Orbitrap mass analyser. The loop cycle was maintained at ten scan per duty cycle. The automatic gain control (AGC) targets for full MS and PRM were at 3E6 and 1E5 ions, respectively, and a maximum of 50 ms for MS1 and 300 ms for PRM scan. For the limit of detection (LOD) experiments (Fig. 1b), the heavy pRab10 peptide was kept at a constant amount of 50 fmol and the light pRab10 peptide varied from (0.01, 0.1, 1, 10, 50 and 200 fmol), directly spiked into 50 ng HeLa cell tryptic digest. PRM data were acquired as described above except the ion injection times for 0.01, 0.1 and 1 fmol data were maintained at 500 ms.

### Spectral library generation

The retention times of heavy stable peptides were determined using an equimolar mixture of 50 fmol and 25 fmol of PRTC peptide mixture was acquired using data dependent acquisition (DDA) mode on QE HF-X mass spectrometer in line with the EvoSep LC system. The DDA data were acquired using one full MS and 10 top N MS2 scans by disabling dynamic exclusion option. MS1 and MS2 scans were acquired at 120,000 and 30,000 resolution at 200 *m*/*z* and measured using Orbitrap mass analyser. Raw data were processed using Proteome Discoverer v2.2 using Sequest search algorithm against Uniprot Human proteome database that was appended with PRTC peptide sequences. Mass error tolerance was set for MS1 at 10 ppm and 0.05 Da for MS2. Carbamidomethylation of Cys as a fixed modification and heavy label (^13^C_6_^15^N_4_) for Arg and (^13^C_6_^15^N_2_) for Lys residues, phosphorylation of Ser/Thr were set as dynamic modification. Percolator node was used to filter that data at 1% FDR. The output.msf files were directly imported into Skyline software suite to serve as a spectral library which was then used for all of the subsequent human neutrophils PRM data.

### MS data analysis and phosphorylation occupancy calculations

All of the PRM raw data were imported into Skyline software suite. An In-house generated spectral library was used to pick the precursor and top six fragment ions. Both double (*z* = 2) and triple (*z* = 3) charge states were used. Extracted ion chromatograms of both MS1 and MS2 fragment transitions were manually examined and adjusted for any interfering ions. The light/heavy internal standard ratio values were exported to calculate the pRab10^Thr73^ occupancy as described before [21]. Briefly, the pRab10^Thr73^ occupancy was calculated by taking the ratio of total amount of phosphorylation to the total amount of both phosphorylated and non-phosphorylated ratio that was represented in terms of percentage, [pRab10 L/H/pRab10 L/H + npRab10 L/H × 100]. All of the neutrophil samples phosphorylation occupancy calculations are provided in Tables 2 and 3.

### MS data availability

All of the proteomics mass spectrometry raw data and database search result output files have been submitted to ProteomeXchange consortium (http://proteomecentral.proteomexchange.org) via the PRIDE repository. The data can be accessed with the following identifier (PXD024898) [36].

### Statistical analysis

Bioinformatic analyses in this study were performed with Skyline (https://skyline.ms/project/home/software/Skyline/begin.view), Microsoft Excel and Graphpad Prism (GraphPad Software). Data visualization was done using GraphPad Prism (GraphPad Software). In general, grouped data analysis was performed using one-way ANOVA for multiple comparisons or unpaired t-test and displayed graphically using GraphPad Prism software (version 9).

## Results

### Clinical cohort and peripheral blood collection

A total of 101 participants were recruited, 66 individuals via the movement disorder clinics at Hospital Clinic Universitari de Barcelona and 35 individuals via the Hospital Universitario Donostia in San Sebastian. Of the 101 participants, 42 carried a pathogenic mutation in LRRK2-21 carried the G2019S mutation that resides in the kinase domain and 21 with the R1441G mutation that lies within the ROC-COR GTPase domain—, 27 patients with iPD, and 32 controls (Table 1). Amongst the LRRK2 mutation carriers were individuals with and without PD. In keeping with the age-dependent penetrance of LRRK2, non-manifesting mutation carriers tended to be younger than those with PD. All participants were clinically evaluated for PD motor symptoms and motor complications using the Unified Parkinson’s Disease Rating Scale (UPDRS) parts III and IV and age at onset of PD and PD duration as well as L-dopa equivalent daily dose (LEDD) calculated where applicable (Supplementary table 2, online resource). 20 ml of fresh peripheral blood was collected for immediate neutrophil isolation followed by ex vivo neutrophil treatment with and without the specific LRRK2 kinase inhibitor MLi-2 (200 nM, 30 min) prior to cell lysis and snap freezing for storage at − 80 °C.

**Table 1:** Summary of demographic and clinical characteristics of participants (*n* = 101)

	LRRK2 mutation carriers (*n* = 42)				iPD	Controls
	G2019S (*n* = 21)		R1441G (*n* = 21)			
	Clinical manifestation (PD/NMC)					
	G2019S-PD	G2019S-NMC	R1441G-PD	R1441G-NMC		
	(*n* = 12)	(*n* = 9)	(*n* = 13)	(*n* = 8)	(*n* = 27)	(*n* = 32)
Age, y	53 (33, 73)	51 (37,69)	59 (42, 78)	58 (49, 85)	68 (53, 80)	60 (40, 82)
Age at onset, y	55 (33, 81)	–	60 (44, 78)	–	60 (37, 78)	–
Disease duration, y	9	–	9	–	8	–
UPDRS Part III (on)	17	–	22	–	19	–
UPDRS IV	4	–	2	–	2	–
LEDD (mg)	580	–	597	–	738	–

Of the 101 participants, 42 carried either the LRRK2 kinase domain mutation G2019S or the ROC-COR GTPase domain mutation R1441G and were either diagnosed with PD or non-manifesting mutation carriers (NMC). Mean age in years (y) and age range in parenthesis, mean disease duration in years with range in parenthesis

*UPDRS* Unified Parkinson’s Disease Rating Scale, part III (motor symptoms) in the on state and part IV (motor complications), *LEDD* l-dopa equivalent daily dosage in mg

All procedures were performed in compliance with the local ethics review boards and all participants provided informed consent.

### Experimental design and workflow

The experimental design and statistical analysis for each experiment are described in each subsection and figure legend. Briefly, all peripheral blood neutrophil samples isolated from participants were subjected to duplicate quantitative multiplex immunoblotting as well as mass-spectrometry with technical replicates per analysis (≥ 2) (Fig. 1). Analysis was performed blinded—in case of the quantitative immunoblotting by independent analysis and in case of the mass-spectrometry with genotype and clinical status only revealed after the mass-spectrometry measurements were completed. To demonstrate that the phosphorylation of Rab10 at Threonine 73 was mediated by the LRRK2 kinase, neutrophils were treated with and without the specific LRRK2 kinase inhibitor MLi-2.

### Sensitivity and specificity of the LRRK2 inhibitor MLi-2 in human peripheral blood neutrophils

The aim for using the LRRK2 kinase inhibitor MLi-2 at a concentration of 200 nM was to achieve near complete dephosphorylation of Rab10 at the LRRK2 dependent phosphoepitope Threonine 73. Dose–response studies of MLi-2 in human peripheral blood neutrophils derived from two healthy donors demonstrated as before [11] that 50% reduction in phosphorylation was achieved at a dose of 30 nM and a suppression of Rab10 phosphorylation to near basal levels at doses of 100 nM and above (suppl. Figure 1, online resource). A similar dose response was previously observed using another phospho-antibody with broader specificity for LRRK2 dependent phosphorylation of the endogenous Rab proteins Rab8A, Rab10 and Rab35 [23]. Additionally, we show that MLi-2 at a concentration of up to 300 nM does not result in dephosphorylation of Rab7a, the endogenous phosphorylation substrate of the only known LRRK2 homologue LRRK1 [25] or dephosphorylation of AMPK at threonine 172. Serine 935 dephosphorylation of LRRK2 is seen at maximal levels starting at 100 nM MLi-2 and above. Total levels of LRRK2, LRRK1 and AMPK did not change (suppl. Figure 1, online resource).

### LRRK2 R1441G mutation carrier status significantly augments LRRK2 dependent pRab10^Thr73^ phosphorylation in neutrophils derived from PD manifesting and non-manifesting individuals

**Fig. 2:**
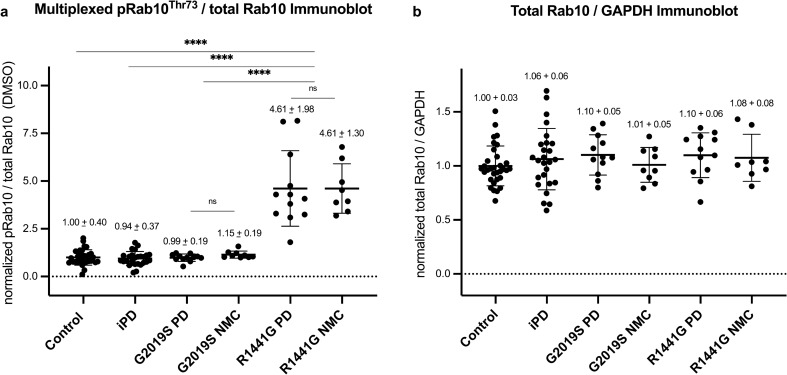
**LRRK2 R1441G mutation carrier status significantly augments LRRK2 dependent pRab10**^**Thr73**^**phosphorylation in neutrophils derived from PD manifesting and non-manifesting individuals**. **a** Grouped analysis of pRab10^Thr73^ phosphorylation levels obtained by multiplexed immunoblotting with *MJFF*-*pRAB10* monoclonal antibodies against the LRRK2 phosphorylated Rab10 phosphoepitope Threonine 73 normalized to the total Rab10 protein levels of DMSO treated neutrophil lysates and to the average of the respective controls. Bars depict group means and standard deviation (SD). Quantifications were based on the average value of at least two independent immunoblot runs with duplicate loading. The LRRK2 mutation carrier groups were further broken down by clinical disease status—either PD manifesting (PD) or non-manifesting carriers (NMC). Differences between groups were calculated by one-way ANOVA followed by multiple comparisons where the mean of each column was compared against the mean of the control group. ****p < 0.0001. **b** Total Rab10 protein levels normalized against the housekeeping protein GAPDH (mean and SD)

We analysed LRRK2 dependent phosphorylation of pRab10^Thr73^ in peripheral blood neutrophils from 42 LRRK2 mutation carriers in comparison to 32 controls as well as 27 iPD patients. Peripheral blood neutrophils were isolated from fresh blood by immunomagnetic negative selection as previously described [12]. Prior to cell lysis neutrophils were split in half and treated with and without the specific LRRK2 kinase inhibitor MLi-2 to demonstrate that pRab10^Thr73^ phosphorylation is mediated by the LRRK2 kinase. When analysed by quantitative multiplex immunoblotting, we observed a striking 4.5-fold increase in LRRK2 dependent pRab10^Thr73^ phosphorylation in R1441G mutation carriers compared to controls, iPD and G2019S mutation carriers (Fig. 2a). When segregated according to clinical disease status, pRab10^Thr73^ phosphorylation status was equally augmented in PD manifesting as well as non-manifesting individuals with R1441G positive mutation status (Fig. 2a and Tables 2, 3). There was no statistically significant difference in pRab10^Thr73^ phosphorylation levels in G2019S mutation carriers irrespective of disease status and also not in iPD patients when compared to controls or with each other. Total Rab10 levels were remarkable consistent amongst participants and did not differ between the groups (Fig. 2b). A representative result of one technical replicate with duplicate loading for both DMSO and MLi-2 treated samples of the immunoblot analysis of all 101 participants is shown in Supplementary Fig. 2, online resource. This also clearly shows the LRRK2 dependency of the pRab10^Thr73^ phosphorylation signal with significant reduction in the LRRK2 kinase inhibitor treated samples of all participants when compared to DMSO.

**Table 2:** Quantification of pRab10^Thr73^ phosphorylation levels by immunoblotting and targeted mass-spectrometry—descriptive analysis of multiplexed immunoblotting of pRab10^Thr73^/total Rab10 ratio normalized to healthy control DMSO neutrophil samples of at least two independent experiments with duplicate loading (≥ 4 data points per sample) and targeted mass-spectrometry measuring absolute pRab10^Thr73^ occupancy (%) of two independent measurements including mean, standard deviation (SD), minimum and maximum for control, iPD and LRRK2 G2019S and R1441G mutation carriers

Multiplex immunoblotting normalized pRab10/total Rab10 (DMSO)	Controls (n = 32)	iPD (*n* = 26)	G2019S (*n* = 21)	R1441G (*n* = 20)
Mean (ratio)	1.00	0.94	1.06	4.61
SD	0.40	0.37	0.20	1.70
Minimum	0.11	0.21	0.53	1.80
Maximum	2.01	1.78	1.58	8.16

Targeted mass-spectrometry pRab10 (DMSO) occupancy	(*n* = 32)	(*n* = 26)	(*n* = 21)	(*n* = 20)
Mean (%)	1.52	1.61	1.90	6.60
SD	0.67	0.65	0.40	2.39
Minimum	0.20	0.20	1.01	2.85
Maximum	3.21	3.22	2.61	12.90

Segregation of LRRK2 mutation carriers according to clinical status did not reveal a statistical difference between mutation carriers with and without PD using unpaired *t* testing (PD vs NMC for LRRK2 G2019S and R1441G) but was not included in this table

**Table 3:** Quantification of pRab10^Thr73^ phosphorylation levels by immunoblotting and targeted mass-spectrometry—one-way ANOVA followed by Tukey’s multiple comparisons where the LRRK2 dependent pRab10^Thr73^ phosphorylation signal for each group was compared against all other groups as well as DMSO vs. MLi-2 treated samples per group including adjusted *p* value, mean difference and 95% confidence interval of difference

Tukey’s multiple comparisons test	Multiplex immunoblotting: normalized pRab10/total Rab10				Targeted mass-spectrometry: pRab10 occupancy (%)			
	Summary	Adj. *p* value	Mean diff	95.00% CI of diff	Summary	Adj. *p* value	Mean diff	95.00% CI of diff
Control DMSO vs. iPD DMSO	ns	> 0.9999	0.06	− 0.4656 to 0.5851	ns	> 0.9999	− 0.0872	− 0.7808 to 0.6064
Control DMSO vs. G2019S DMSO	ns	> 0.9999	− 0.06	− 0.6140 to 0.5036	ns	0.7636	− 0.3795	− 1.117 to 0.3583
Control DMSO vs. R1441G DMSO	****	< 0.0001	− 3.61	− 4.177 to − 3.043	****	< 0.0001	− 5.073	− 5.822 to − 4.325
iPD DMSO vs. G2019S DMSO	ns	1.00	− 0.11	− 0.6987 to 0.4689	ns	0.9414	− 0.2923	− 1.063 to 0.4785
iPD DMSO vs. R1441G DMSO	****	< 0.0001	− 3.67	− 4.262 to − 3.078	****	< 0.0001	− 4.986	− 5.768 to − 4.205
G2019S DMSO vs. R1441G DMSO	****	< 0.0001	− 3.56	− 4.177 to − 2.933	****	< 0.0001	− 4.694	− 5.515 to − 3.873
Control (DMSO vs. MLi-2)	ns	> 0.9999	0.00	− 0.4975 to 0.4975	****	< 0.0001	1.224	0.5669 to 1.880
iPD (DMSO vs. MLi-2)	ns	> 0.9999	− 0.02	− 0.5675 to 0.5362	****	< 0.0001	1.361	0.6251 to 2.097
G2019S (DMSO vs. MLi-2)	ns	> 0.9999	− 0.03	− 0.6421 to 0.5861	****	< 0.0001	1.626	0.8158 to 2.437
R1441G (DMSO vs. MLi-2)	****	< 0.0001	3.62	2.994 to 4.252	****	< 0.0001	6.331	5.490 to 7.173

For the immunoblotting analysis it is important to note that results are normalized against the means of controls for the three separate sets of experiments as highlighted in the method section

*ns* non-significant

### Positive correlation between Western blotting and targeted mass-spectrometry assays for LRRK2 dependent pRab10^Thr73^ phosphorylation

In parallel to quantitative immunoblotting for pRab10^Thr73^ phosphorylation, the neutrophil samples were subjected to an ultra-sensitive targeted mass-spectrometry (MS) based assay for analysing pRab10^Thr73^ phosphorylation occupancy with spike in of synthetic stable isotope-labelled (SIL) tryptic Rab 10 peptides around the Threonine 73 epitope in its phosphorylated as well as unphosphorylated form as internal standards [21] (Fig. 1). This analysis allowed us to address reproducibility of our immunoblotting results by an independent method, correlation between the two assays and importantly explore whether a potentially more sensitive analysis by mass-spectrometry could yield a statistically significant difference in pRab10^Thr73^ phosphorylation levels for the G2019S mutation carrier group or iPD patients when compared to controls.

**Fig. 3:**
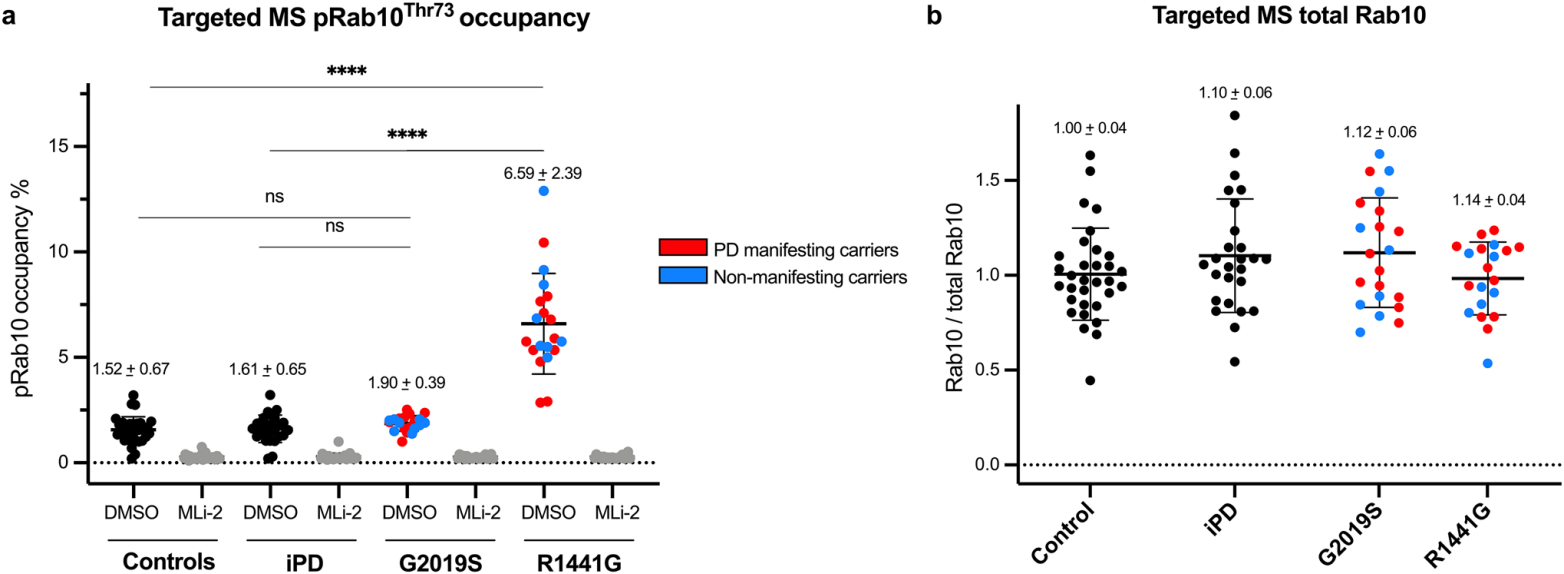
**Significantly elevated pRab10**^**Thr73**^**phosphorylation occupancy in neutrophils derived from R1441G mutation carriers with and without PD**. **a** Quantification of pRab10^Thr73^ occupancy (%) in DMSO and MLi-2 treated neutrophil lysates derived from controls, iPD and LRRK2 mutation carriers of either the G2019S or R1441G mutation. PD or non-manifesting carriers (NMC) status indicated in DMSO samples by colour. One-way ANOVA with multiple comparisons was applied with the mean of each column being compared with the mean of the control group. pRab10^Thr73^ occupancy is presented as means ± SD. ****p < 0.000. There was no statistically significant difference between manifesting and NMC carriers for both R1441G and G2019S mutations (see also Table 2). **b** Peak areas of endogenous total Rab10 (two peptides) were summed and normalized to median intensity displayed with mean and SD. Proteomic analysis of total Rab10 peptide levels did not differ between groups

We measured pRab10^Thr73^ phosphorylation occupancies in all MLi-2 treated and untreated (DMSO) neutrophil samples from all 101 participants (Fig. 3a). Consistent with immunoblotting data, the average pRab10^Thr73^ occupancy in the control group was 1.52% whereas it increased over fourfold to 6.60% in the R1441G mutation carrier group (*p* < 0.0001, one-way Anova). Here again, the MS assay confirmed that there was no significant difference between control group occupancies in comparison to the iPD (1.61%) and G2019S mutation carrier groups (1.90%) (Tables 2, 3). Furthermore, when LRRK2 mutation carriers were separated by clinical PD status and analysed by unpaired t-test there was also no difference between R1441G-PD manifesting and non-manifesting individuals (*p* = 0.23) or between G2019S-PD manifesting and non-manifesting individuals (*p* = 0.33) (Fig. 3a and Table 3). Figure 3a also shows the respective pRab10^Thr73^ phosphorylation occupancies in the LRRK2 kinase inhibitor treated samples (MLi-2) which was significantly reduced when compared to the untreated (DMSO) samples for each group (*p* < 0.0001, Table 3) and, therefore, clearly demonstrating that the phosphorylation of pRab10^Thr73^ is mediated by the LRRK2 kinase. As with the analysis by Western blotting, mass-spectrometry analysis for total Rab10 levels did not significantly differ between the groups (Fig. 3b).

**Fig. 4:**
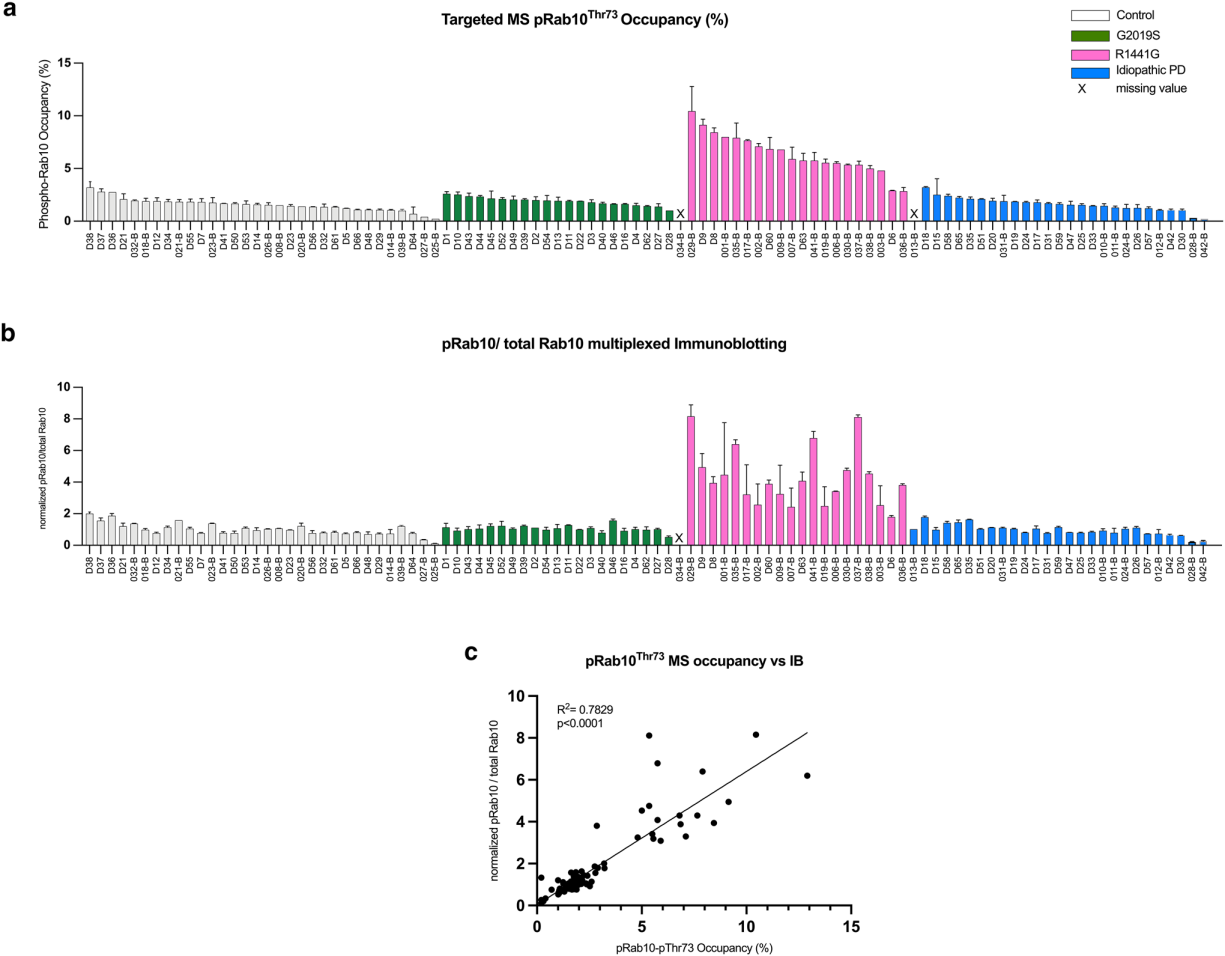
**Good correlation between quantitative multiplexed phosphorylated Rab10**^**Thr73**^**/total Rab10 immunoblotting and targeted pRab10**^**Thr73**^**occupancy assays in peripheral blood neutrophil﻿s**. **a** pRab10^Thr73^ phosphorylation occupancies in DMSO treated neutrophils per individual arranged in descending order by group (**b**) and aligned quantitative immunoblotting analysis of pRab10^Thr73^/total Rab10 ratios (**b**) were plotted as mean with SD for all 101 participants. **c** Pearson correlation between immunoblotting for pRab10^Thr73^ phosphorylation/total Rab10 protein levels and targeted pRab10^Thr73^ occupancies. Blotted are mean values of two independent experiments for each method (*R*^2^ = 0.78, *p* < 0.0001)

**Fig. 5:**
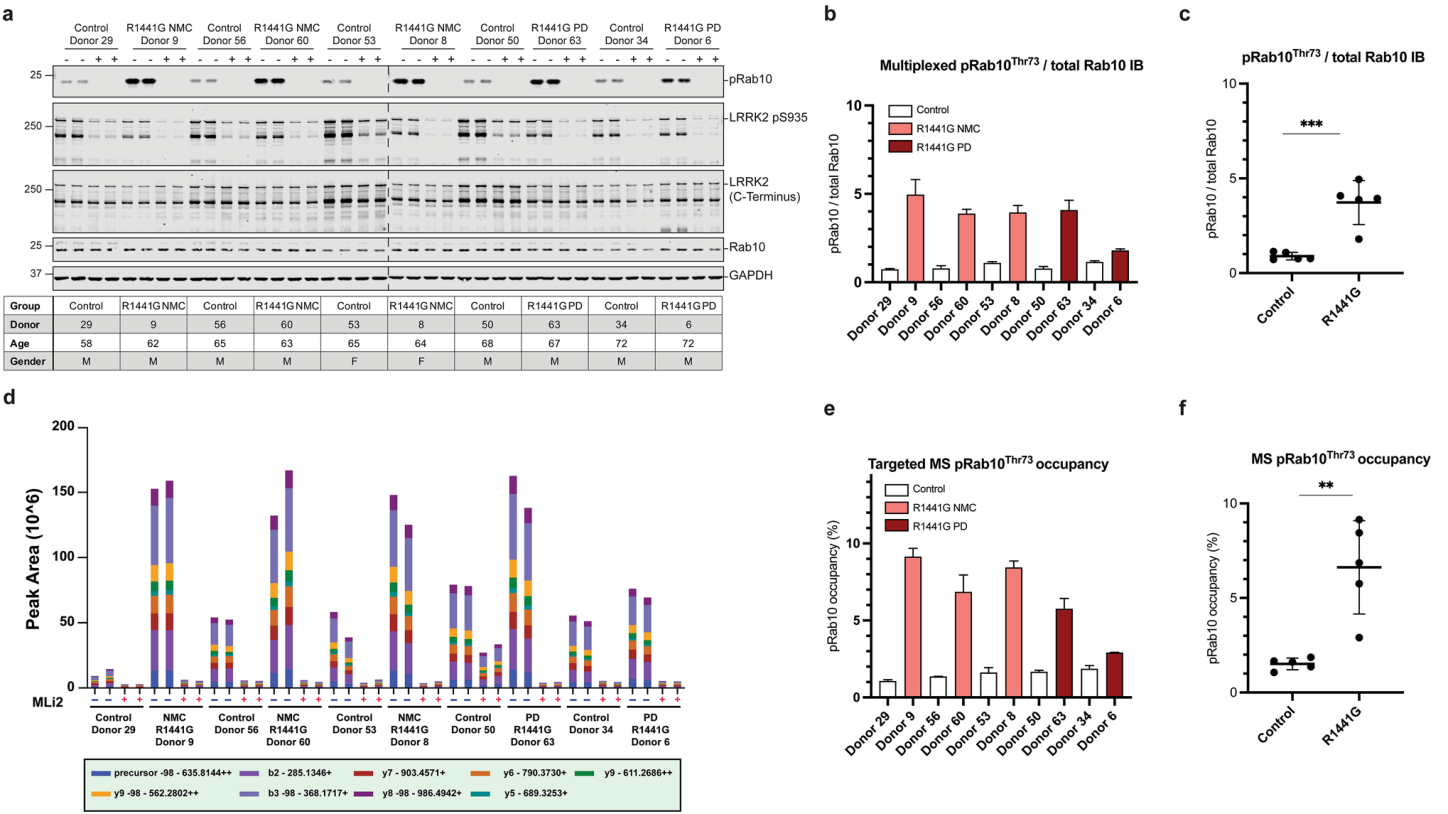
**Representative results for five controls and five R1441G mutation carriers**. **a** Immunoblotting of neutrophils isolated from five R1441G LRRK2 mutation carriers with PD (*n* = 2) and non-manifesting carrier status (*n* = 3) as well as five controls with duplicate loading of 10 µg of DMSO and MLi-2 (200 nM MLi-2, 30 min) treated whole cell extracts using antibodies against total LRRK2, pSer935 LRRK2, total Rab10, MJFF-pRAB10 (pThr73) and GAPDH. (b) Quantification of immunoblots of two independent experiments by analysing pRab10^Thr73^ /total Rab10 ratio of DMSO treated samples **b** per individual and **c** per group with group difference calculated by unpaired t test (***p = 0.0007). **d** The respective summed intensities of fragment ion transitions are represented with different colours as shown in the bottom panel of the graph. **e** Relative endogenous pRab10 peak areas of two independent analysis for pRab10^Thr73^ occupancy depicted as a bar graph for DMSO and MLi-2 treated samples of all ten participants and **f** per group with group difference calculated by unpaired *t* test (**p = 0.0014)

While the targeted pRab10^Thr73^ phosphorylation mass-spectrometry analysis of our neutrophil samples independently confirmed that neutrophils from LRRK2 R1441G, but not G2019S mutation carriers or patients with iPD have significantly elevated LRRK2 dependent Rab10 phosphorylation levels, we were interested in directly comparing the results obtained by the two methods. We, therefore, aligned the MS and WB data per individual (Fig. 4a, b) and found good correlation between the two methods (*R*^2^ = 0.78, *p* < 0.0001) (Fig. 4c). Representative results are shown in Fig. 5 where duplicate DMSO and MLi-2 treated neutrophil samples from the same 10 individuals including five controls and five R1441G mutation carriers were analysed for pRab10^Thr73^ phosphorylation levels by quantitative Western blotting (Fig. 5a) and targeted pRab10^Thr73^ mass-spectrometry (Fig. 5b). The data also clearly show that the pRab10^Thr73^  phosphorylation signal was significantly reduced to almost undetectable levels when neutrophils were treated with the specific LRRK2 kinase inhibitor MLi-2 confirming the specificity of the pRab10^Thr73^ phosphorylation signal as a substrate of the LRRK2 kinase in this cell type.

### Biological sex did not impact on LRRK2 dependent pRab10^Thr73^ phosphorylation

As male and female sex is an important determinant for PD susceptibility, we assessed the effect of sex on LRRK2 dependent pRab10^Thr73^ phosphorylation in neutrophils. Overall, there was no significant difference in pRab10^Thr73^ phosphorylation occupancies between male and female participants (data not shown) and also no significant difference when each group including controls, iPD, G2019S and R1441G mutation carriers was segregated by sex (Supplementary Fig. 4, online resource).

### LRRK2 total protein and LRRK2 Serine 935 phosphorylation levels in peripheral blood neutrophils

**Fig. 6:**
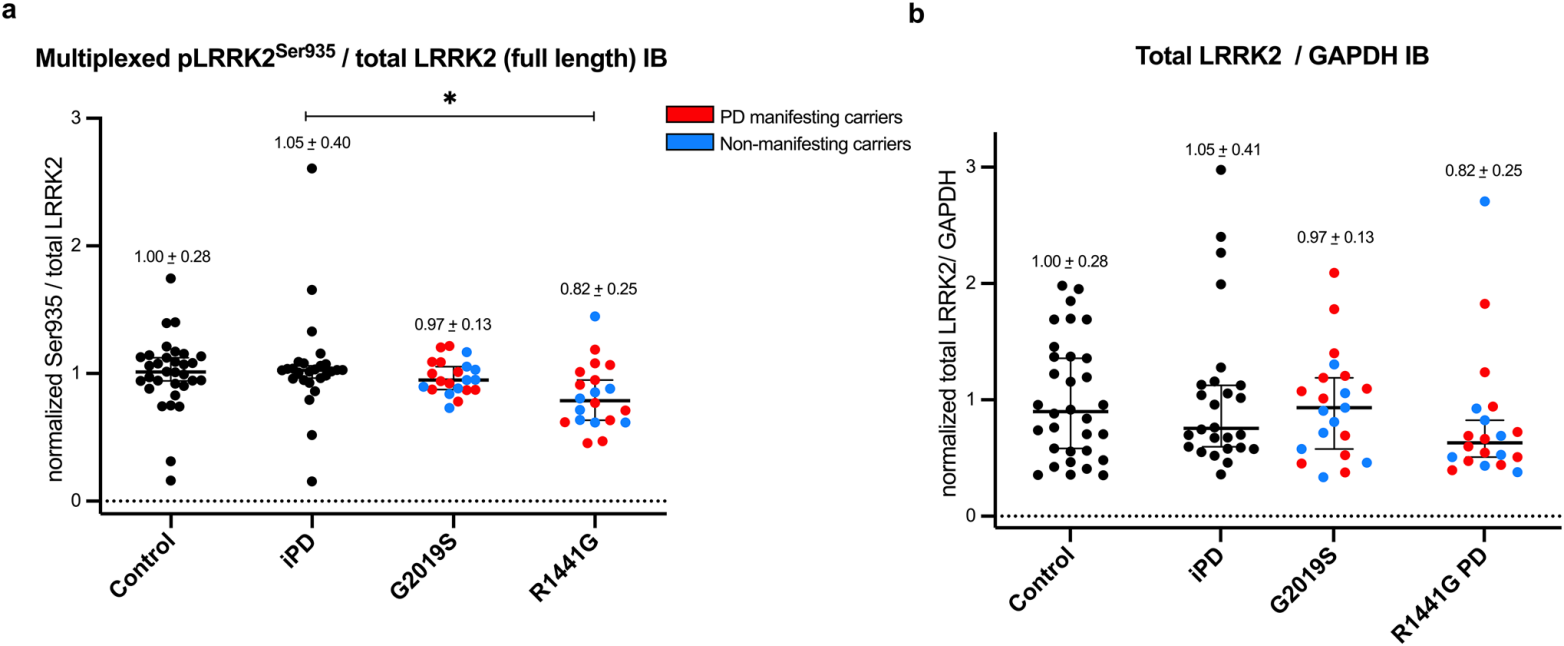
**Serine 935 phosphorylation of LRRK2 and total LRRK2 levels in peripheral blood neutrophils.** Normalized multiplexed pSerine935/total LRRK2 protein ratio (**a**) and normalized total LRRK2/GAPDH protein levels (**b**) of DMSO treated neutrophil lysates per group. Bars depict mean values with SD. Quantifications were based on the average value of two independent immunoblot runs. Differences between groups were calculated by one-way ANOVA followed by multiple comparisons where the mean of each column was compared against the mean of the other groups. There was a small, but statistically significant difference between the R1441G mutation carrier and iPD groups *p = 0.0454 for pSerine 935/total LRRK2 while total LRRK2/GAPDH ratio remained largely unchanged between groups. When segregated by disease status, there was no significant difference between PD manifesting and non-manifesting mutation carriers for G2019S and R1441G (data not shown). For LRRK2 and pSerine935 quantification the higher molecular weight band representing full length LRRK2 was used

In addition to phosphorylated and total Rab10 levels, we analysed Serine 935 phosphorylation of LRRK2 and total LRRK2 levels by quantitative Western blotting in all peripheral blood neutrophil samples. We observed a small, but statistically significant reduction in Serine 935 phosphorylation of LRRK2 in R1441G mutation carriers when compared to iPD (*p* = 0.045), but no difference when compared to G2019S mutation carriers or controls (Fig. 6a). This is consistent with previous work that has revealed that the R1441G mutation reduces Serine 935 phosphorylation [33, 40]. When Serine 935 phosphorylation levels were compared between R1441G mutation carriers with and without PD, there was no difference (*p* = 0.99). We also did not find a statistically significant reduction of Serine 935 phosphorylation of LRRK2 in G2019S mutation carriers irrespective of disease status when compared to iPD or in fact any of the other groups. LRRK2 total protein levels did not significantly differ between the R1441G mutation carriers, G2019S mutation carriers, iPD and controls (Fig. 6b).

### pRab10^Thr73^ phosphorylation in human post-mortem brain samples

**Fig. 7:**
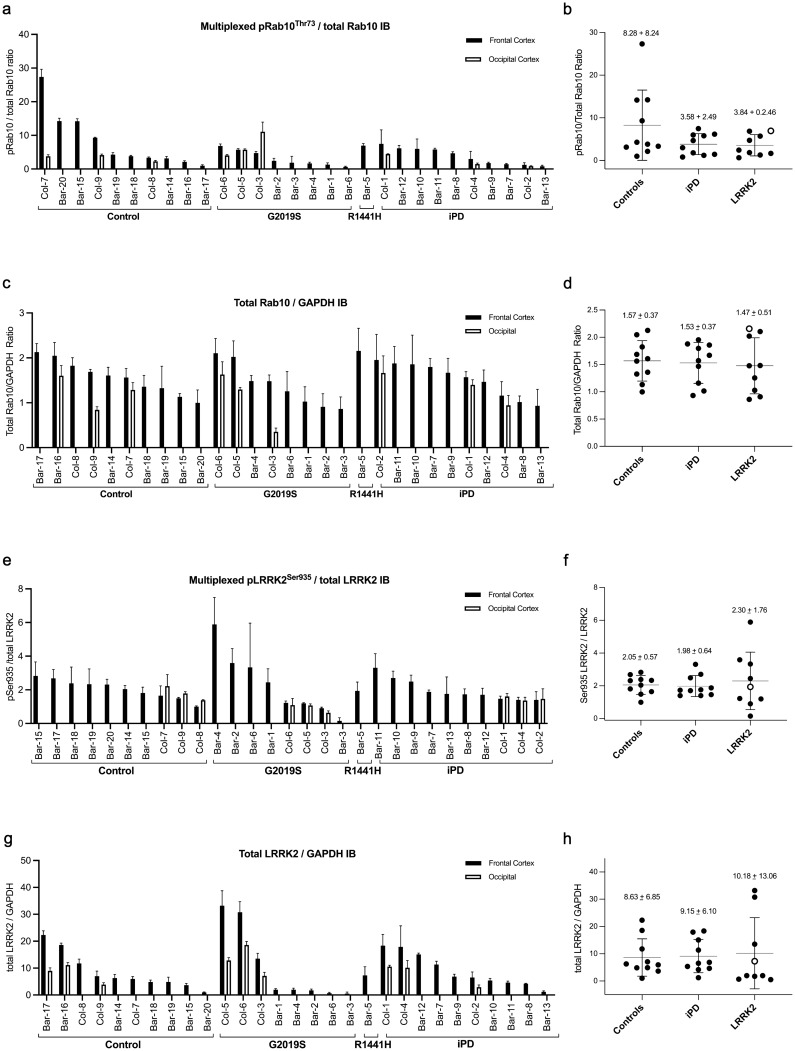
**Quantification of pRab10**^**Thr73**^**phosphorylation levels in post-mortem brain samples from LRRK2 mutation carriers, iPD and controls**. Quantifications were based on the average value of two independent Western blotting experiments with duplicate sample loading. Quantification of phosphorylated pRab10^Thr73^/total Rab10 ratio and total Rab10 protein levels/GAPDH per sample with columns indicating means and SD (**a**, **c**) and per group for the frontal cortex samples (**b**, **d**), respectively. The same analysis was performed for LRRK2 Serine935 phosphorylation/total LRRK2 protein ratio and total LRRK2/GAPDH protein levels per sample (**e**, **g**) and per group for the frontal cortex samples (**f**, **h**), respectively. The R1441H mutation carrier sample is marked with a white circle with black rim in the grouped analysis. Overall, there were no significant group differences

PD is considered a condition of the central nervous system and an important question is how the analysis of LRRK2 dependent pRab10^Thr73^ phosphorylation in human peripheral blood neutrophils compares to the analysis of pRab10 ^Thr73^ phosphorylation and LRRK2 levels in the brain. To address this, we obtained matched frontal and occipital cortex samples from 9 individuals including 3 controls, 3 G2019S mutation carriers with PD and 3 iPD from the brain bank at the Columbia University Medical Center in New York. Additionally, we were able to access 20 frontal cortex samples including 7 controls, 7 iPD, 5 G2019S and 1 R1441H mutation carriers with PD from the IDIBAPS Biobank at the Hospital Clinic in Barcelona, Spain. Demographic and clinical information is provided in Supplementary table 3, online resource. We used multiplexed quantitative immunoblotting (suppl. Figure 4, online resource) to assess pRab10^Thr73^ phosphorylation against total Rab10 levels and LRRK2 Serine 935 phosphorylation against total LRRK2. Total protein levels for Rab10 and LRRK2 were measured by normalization against a housekeeping protein. With regards to pRab10^Thr73^ phosphorylation, we observed high variability amongst samples from low to almost undetectable levels in the majority of samples and very high levels in some irrespective of group or LRRK2 mutation status; in fact, some of the highest pRab10^Thr73^ phosphorylation levels were observed amongst the control samples. Also, the LRRK2 R1441H genotype, which is as R1441G another hotspot mutation at the R1441 site and known to significantly augment LRRK2 dependent pRab10^Thr73^ phosphorylation in the heterologous overexpression system (unpublished results), did not result in significantly elevated pRab10^Thr73^ phosphorylation levels. While the variances were higher for the control samples (standard deviation = 8.233 vs. 2.495 (LRRK2 mutation carriers) and 2.640 (iPD)), there were no significant differences between the control, LRRK2 mutation carrier and iPD groups (Fig. 7b). We also tested whether biological sex had an overall effect on pRab10^Thr73^ phosphorylation, but there was no significant difference between frontal cortex samples derived from female or male donors (data not shown). Total Rab10 levels displayed less variability amongst frontal cortex samples and did not significantly differ between the groups (Fig. 7d). There was also a high degree of variability in Serine 935 phosphorylation levels of LRRK2 (Fig. 7f) and total LRRK2 levels (Fig. 7h) amongst samples, but overall, no significant difference between the LRRK2 mutation carrier, iPD and control groups. When comparing frontal and occipital lobe samples where matched pairs were available, pRab10^Thr73^ phosphorylation tended to be lower in the occipital lobe than in the frontal lobe (with the exception of one G2019S mutation carrier (Col-3) where the levels in the occipital lobe were higher) (Fig. 7a) and total Rab10 levels were always lower in the occipital lobe samples (*p* = 0.0016) (Fig. 7c). For Serine 935 phosphorylation of LRRK2 (Fig. 7e), there was no significant difference between matched frontal and occipital lobe samples (paired t test, *p* = 0.3235), while total LRRK2 levels were significantly reduced in the occipital lobe when compared to matched frontal lobe samples (*p* = 0.001) (Fig. 7g). Additionally, we subjected the brain lysates to immunoblotting for the PPM1H phosphatase that counteracts LRRK2 signalling by selectively dephosphorylating Rab proteins including Rab10 [2], but did not observe significant changes amongst samples or groups except for reduced levels in sample Col-3 (suppl. Figure 4, online resource).

## Discussion

This is to date the largest study of LRRK2 dependent phosphorylation of its endogenous substrate pRab10^Thr73^ in peripheral blood neutrophils in LRRK2 mutation carriers, controls and individuals with iPD. Our results include four key observations: (1) We show for the first time that LRRK2 dependent pRab10^Thr73^ phosphorylation as a surrogate marker for LRRK2 kinase activation status is significantly elevated in carriers of a specific pathogenic variant in the LRRK2 gene, namely the R1441G mutation in the ROC-COR GTPase domain. (2) This significant increase in pRab10^Thr73^ phosphorylation in LRRK2 R1441G mutation carriers is irrespective of clinical disease status and observed in non-manifesting carriers as well as those diagnosed with PD. (3) We have not observed a statistically significant increase in LRRK2 dependent pRab10^Thr73^ phosphorylation in carriers of the common G2019S mutation deploying two independent assays. (4) Furthermore, our explorative analysis of markers of the LRRK2 kinase pathway in human autopsy brain samples highlights important aspects and indeed problems with regards to feasibility, suitability and differences across different biomatrices. Additionally, we show that both Serine 935 phosphorylation of LRRK2 as well as pRab10^Thr73^ phosphorylation serve as biomarkers for LRRK2 kinase inhibition when using kinase inhibitors such as MLi-2. In this regard, it is important to note that pharmacokinetic properties vary depending on cellular context and assay readouts and that a relatively high concentration of MLi-2 was required to suppress Rab10 phosphorylation at Threonine 73 in peripheral blood neutrophils compared to previously reported IC50 of between 0.76 nM and 3.4 nM [11, 13].

Utilizing our facile and robust multiplex immunoblotting assay using sensitive phosphospecific antibodies against the Threonine 73 phospho-site of Rab10 that is phosphorylated by the LRRK2 kinase, a statistically significant increase of more than fourfold in pRab10^Thr73^ phosphorylation levels was seen in LRRK2 R1441G mutation carriers when compared to iPD, G2019S mutation carriers or controls. This increase was irrespective of disease status, and there was no significant difference between R1441G mutation carriers with and without PD manifestation. We also did not see an effect of female or male sex on LRRK2 dependent pRab10^Thr73^ phosphorylation. This suggests that the effect on pRab10^Thr73^ phosphorylation levels in circulating blood neutrophils is mainly driven by the underlying mutation itself rather than by the accompanying PD disease process. In future experiments it would be interesting to monitor larger numbers of participants and over time to better assess the utility of LRRK2 dependent pRab10^Thr73^ phosphorylation as a marker of LRRK2 driven disease conversion or disease state.

Heterologous systems such as HEK293 overexpression of LRRK2 variants and genetic animal models with knock-in of LRRK2 mutations in the homozygous state have shown that the LRRK2 ROC and COR GTPases domain mutations—including R1441G/H that suppress GTPases activity and promote GTP binding—mediate a 3 to 4-fold increase in LRRK2 kinase activity [38, 39] while LRRK2 kinase domain mutations such as G2019S increase LRRK2 kinase activity only modestly, typically under twofold (reviewed in [40]) by domain disruption. In our experiments, we did not observe a statistically significant increase in pRab10^Thr73^ phosphorylation levels in 21 LRRK2 G2019S mutation carriers despite promising results from a previous much smaller study of pRab10^Thr73^ phosphorylation stoichiometry in neutrophils from 4 G2019S mutation carriers in comparison to 4 non-mutation carriers [21]. While there was good correlation between our two assays which allowed for independent confirmation of our results, the potentially more sensitive mass-spectrometry assay did not yield any additional information with regards to LRRK2 dependent pRab10^Thr73^ phosphorylation levels. Given that the LRRK2 G2019S mutation induces an under twofold enhancement of pRab10^Thr73^ phosphorylation in homozygosity, one would expect a relative reduction to < 1.5-fold in heterozygosity. We, therefore, conclude that the effect of the LRRK2 G2019S mutation in heterozygosity as with the participants in our study coupled with the biological variation in between human samples is too small to accurately yield a significant difference by quantitative multiplexed immunoblotting or even more sensitive mass-spectrometry methodologies. On the other hand, measuring pRab10^Thr73^ phosphorylation levels using our relatively straightforward immunoblotting assay [12] may be attractive for researchers without access to a mass-spectrometry facility as the data obtained was similar in quality and resolution to the mass-spectrometry data.

A recent study found a statistically significant decrease in Serine 935 phosphorylation of LRRK2 in human peripheral blood mononuclear cells (PBMCs) derived from LRRK2 G2019S mutation carriers with PD but not G2019S non-manifesting mutation carriers when compared to iPD [35]. Here, we have not observed a reduction in Serine 935 phosphorylation levels in G2019 mutation carriers with PD in comparison to iPD or in fact in comparison to any of the other groups. However, the differences in biomatrix—PBMCs vs neutrophils—and assay methodology—digital immunoassay vs quantitative multiplexed immunoblotting—may account for this discrepancy. For example, neutrophils represent a homogenous cell population with high LRRK2 expression, but their intrinsically very active serine proteases appear to affect disproportionately high molecular weight species such as LRRK2 (288 kDa) with resulting partial LRRK2 degradation [11]. This particular problem could be addressed using peripheral blood monocytes for LRRK2 pathway analysis as described by us previously [27]. It would, therefore, be of interest to expand and compare Serine 935 phosphorylation of LRRK2 across different peripheral blood cell populations including monocytes and additional patient cohorts. What we did observe was a reduction in Serine 935 phosphorylation in LRRK2 R1441G mutation carriers when compared to iPD, but not when segregated by clinical status or in comparison to LRRK2 G2019S mutation carriers or controls, a finding that is consistent with previous studies [9, 40]. Importantly, our results also clearly show that LRRK2 Serine 935 phosphorylation is not a marker of LRRK2 kinase activity.

pRab10^Thr73^ phosphorylation has previously been shown to be elevated in substantia nigra of patients with iPD when compared to controls [8]. While we were interested in extending our analysis to post-mortem brain samples of LRRK2 mutation carriers and iPD in comparison to controls and assess its correlation with peripheral blood neutrophils, we were unable to obtain substania nigra samples, but only frontal and additionally occipital cortex samples in some. While total Rab10 protein levels were relatively stable amongst all samples, the pRab10^Thr73^ phosphorylation signal was not or hardly detectable in the majority of cases and otherwise variable irrespective of genotype and clinical disease status. In particular, we did not observe significant pRab10^Thr73^ hyperphosphorylation in the one LRRK2 R1441H mutation carrier sample which biochemically should have the same augmenting effect on LRRK2 dependent pRab10^Thr73^ phosphorylation as the R1441G mutation [39]. While the LRRK2 total protein and Serine 935 phosphorylation of LRRK2 signals could be detected there was again a large degree of disparity for Serine 935 phosphorylation between samples. Overall, the main finding was that there was high variability amongst individual samples in all parameters that we analysed, and this is consistent with our previous study of human brain cingulate cortex samples from 23 controls and 28 individuals with iPD [24].

While it would be worthwhile to expand the analysis of pRab10^Thr73^ phosphorylation in autopsy brain material to a larger number of cases, in particular of carriers of R1441 hotspot or the VPS35 D620 mutations, there are important factors to consider such as circumstances and cause of death and post-mortem interval that may affect tissue quality and in particular stability of posttranslational phosphorylation modifications [22]. Another concern above and beyond the regional variation of brain tissue is its heterogeneity in terms of cellular composition which is not taken into account in the assay deployed in this study. We, therefore, conclude that measuring pRab10^Thr73^ phosphorylation in post-mortem brain lysates is uninformative mainly due to the instability of posttranslational modifications.

In conclusion our findings add compelling evidence that interrogating in vivo LRRK2 dependent pRab10^Thr73^ phosphorylation in human peripheral blood neutrophils is a specific, robust and promising biomarker for significant LRRK2 kinase hyperactivation, as with the LRRK2 R1441G mutation or as previously demonstrated with the VPS35 D620N mutation [27]. Further work is required to explore additional readouts including phosphorylation levels of other RabGTPase substrates of the LRRK2 kinase [34], assays and biomatrices that would allow detection of more modest LRRK2 kinase activation as with the LRRK2 G2019S mutation and possibly in individuals with iPD. We envision that our assays will be used alongside other markers of the LRRK2 signalling and PD associated pathways across different biomatrices and importantly longitudinally to possibly delineate integrated markers for disease conversion and PD progression.

## Supplementary Information

Below is the link to the electronic supplementary material.

Supplementary file1 (PDF 8801 kb)
