# Supplementary material for: R1441G but not G2019S mutation enhances LRRK2 mediated Rab10 phosphorylation in human peripheral blood neutrophils
Source: Acta Neuropathol. 2021 Jun 14;142(3):475–94. doi: 10.1007/s00401-021-02325-z (PMC8357670; doi:10.1007/s00401-021-02325-z)
Supplement: Supplementary file 1 — Supplementary file1 (PDF 8801 kb) [file 401_2021_2325_MOESM1_ESM.pdf]

R1441G but not G201S mutation enhances LRRK2 mediated Rab10 phosphorylation in human peripheral blood neutrophils

Ying Fan[1], Raja S. Nirujogi[1], Alicia Garrido[2,3], Javier Ruiz Martínez[4], Alberto Bergareche-Yarza[4], Elisabet Mondragón Rezola[4], Ana Vinagre Aragón[4], Ioana Croitoru[4], Ana Gorostidi Pagola[4], Laura Paternain Markinez[4], Roy Alcalay[5], Richard A. Hickman[6], Jonas Duering[1], Sara Gomes[1], Neringa Pratuseviciute[1], Shalini Padmanabhan[7], Francesc Valldeoriola[2,3], Leticia Pérez Sisqués[8], Cristina Malagelada[8,9], Teresa Ximelis[10], Laura Molina Porcel[10,11], Maria José Martí[2,3], Eduardo Tolosa[2,3], Dario R Alessi[1], Esther Sammler*[1,12]

1. Medical Research Council Protein Phosphorylation and Ubiquitylation Unit, University of Dundee, Dundee, DD1 5EH, UK.
2. Parkinson's disease and Movement Disorders Unit, Neurology Service, Hospital Clínic de Barcelona, Barcelona, Spain.
3. Centro de Investigación Biomédica en Red Sobre Enfermedades Neurodegenerativas (CIBERNED), Hospital Clínic, IDIBAPS, Universitat de Barcelona, Barcelona, Spain.
4. Group of Neurodegenerative Diseases, Biodonostia Health Research Institute, San Sebastian, Spain.
5. Department of Neurology, Columbia University Medical Center, New York, NY, USA.
6. Department of Pathology and Cell Biology, Columbia University Medical Center New York, NY, USA.
7. The Michael J Fox Foundation for Parkinson's Research, New York, NY, USA.
8. Departament de Biomedicina, Facultat de Medicina i Ciències de la Salut, Institut de Neurociències. Universitat de Barcelona, Barcelona, Catalonia, Spain.
9. Centro de Investigación Biomédica en Red sobre Enfermedades Neurodegenerativas (CIBERNED), Madrid, Spain.
10. Neurological Tissue Bank of the Biobanc-Hospital Clinic-Institut d'Investigacions Biomediques August Pi i Sunyer (IDIBAPS), Barcelona, Spain.
11. Alzheimer's disease and other cognitive disorders unit. Neurology Service, Hospital Clínic, Institut d'Investigacions Biomediques August Pi i Sunyer (IDIBAPS), University of Barcelona, Barcelona, Spain.
12. Molecular and Clinical Medicine, Ninewells Hospital and Medical School, University of Dundee, DD1 9SY, UK.

Corresponding author*: Esther Sammler, e.m.sammler@dundee.ac.uk, 0044 7545072451 (mobile), 0044 1382 223778 (fax)

Supplementary Table 1. List of materials and reagents. Antibodies (a), chemical reagents (b), synthetic peptides (c).

a

Epitope and host	Source or reference	Catalogue #	Comments
anti-tubulin (mouse monoclonal)	Cell Signaling Technology	3873	WB (1 µg/ml)
anti-total LRRK2 N-terminal antibody (rabbit monoclonal)	MRC PPU Reagents and Services	UDD3	WB (1 µg/ml)
anti-phospho-S935-LRRK2 (rabbit monoclonal)	MRC PPU Reagents and Services	UDD2	WB (1 µg/ml)
anti-C-terminal LRRK2 (mouse monoclonal)	Neuromab	Clone number: N241A/34, #75-253	WB (1 µg/ml)
anti antibody phospho-Rab10 (Thr73) (rabbit monoclonal)	Abcam	MJF-21-108-10 (ab230261)	WB (1 µg/ml)
anti-total Rab10 (mouse monoclonal)	Nanotools	0680–100/Rab10-605B11	WB (0.5 µg/ml)
anti-GAPDH (mouse monoclonal)	Santa Cruz	sc-32233	1:10,000 dilution
Goat anti-mouse IRDye 680LT	LI-COR	926-68020	1:10,000 dilution
Goat anti-mouse IRDye 800CW	LI-COR	926-32210	1:10,000 dilution
Goat anti-rabbit IRDye 800CW	LI-COR	926-32211	1:10,000 dilution
anti-phospho-Rab8 (Thr72) (rabbit monoclonal)	ABCAM	#ab230260	WB (1 µg/ml)
anti-phospho AMPK (Thr172) (rabbit monoclonal)	Cell Signaling Technology	#4188	WB (1 µg/ml)
anti-total AMPK (mouse monoclonal)	Cell Signaling Technology	#2793	WB (1 µg/ml)
anti-Rab7A (mouse monoclonal)	Sigma	#R8779	WB (1 µg/ml)
anti-total LRRK1 (sheep polyclonal)	MRC PPU Reagents and Services	sheep number S405C, 2nd bleed	WB (1 µg/ml)
anti-phospho-Rab7A (Ser72) (rabbit polyclonal)	The MJFF's research tools program / Abcam	tools@michaeljfox.org	WB (1 µg/ml)
anti-PPM1H (sheep polyclonal)	MRC PPU Reagents and Services	DA018	WB (1 µg/ml)

WB=Western blot

b

Chemical Name	Company	Catalogue number	Comment
15 cm Analytical column	EvoSep	EV-1106	
18-gauge syringe needle	Sigma Aldrich	Z261351	
2 cm Trap column	Thermo Fisher Scientific	164562	
50 cm Easy Spray column	Thermo Fisher Scientific	ES803	
8 cm analytical column	EvoSep	EV-1109	
Acetonitrile	VWR	20048.32	
Bovine serum albumin	Sigma Aldrich	5470	
C18 celan up tips	Starstedt	70.760.501	
C18 Sep-Pak cartridges	Waters	WAT051910	
CBQCA	Invitrogene	C6667	
cis-2,6-dimethyl-4-(6-(5-(1-methylcyclopropoxy)-1H-indazol-3-yl)pyrimidin-4-yl)morpholine (MLi-2)		Synthesized in-house by Natalia Shpiro	
Diisopropylfluorophosphate (DIFP)	Sigma Aldrich	D0879	
Direct Human Neutrophil Isolation Kit	Stemcell	19666	
EasySep Magnet	Stemcell	18002	
Empore C18 disks	CDS-Analytical	2215	https://www.cdsanalytical.com
EvoTips	EvoSep	EV2001	
Evotips	EvoSep	EV2001	
ExpiFectamine™ 293 Transfection Kit	Thermo Fisher Scientific	A14525	
Formic acid	Sigma Aldrich	695076	
Iodoacetamide	Sigma Aldrich	I1149	
KCN	Sigma Aldrich	60178	
Methanol	VWR	1.06035.2500	
Microcystin-LR	Enzo	ALX-350-012-M001	
MOPS	Sigma Aldrich	69947	
Phosphate buffered saline	Gibco	10010023	
PRTC-Mix	Pierce, Thermo	88320	
S-Trap mini columns	Protifi	S-Trap mini CO2-mini-80	https://www.protifi.com/
Sodium Fluoride	Sigma Aldrich	S7920	
Sodium orthovandate	Sigma Aldrich	S6508	
Sodium phosphate dibasic	Sigma Aldrich	S9763	
Sodium pyrophosphate	Sigma Aldrich	P8010	
TPCK treated trypsin	Sigma Aldrich	T1426-100MG	
Triethyl ammonium bicarbonate buffer	Sigma Aldrich	T7408	
Trifluroacetic acid	Sigma Aldrich	302031	
Tris(2-carboxyethyl)phosphine hydrochloride (TCEP)	Sigma Aldrich	C4706	
Urea Sequenal grade	Thermo Fisher Scientific	29700	
β-Glycerophosphate disodium salt hydrate	Sigma Aldrich	G5422	
Trypsin	Promega	V511A	
cOmplete(EDTA-free) protease inhibitor cocktail	Roche	11836170001	
EasySep Direct Human Neutrophil Isolation Kit	Stemcell	19666	

c

Gene name	Phosphopeptide (* denotes heavy amino acid)	PTM modification	Species	Phosphosite position (Mouse)	Phosphosite position (Human)	Heavy/Light label	Charge	MH+	m/z (*z*=2)
RAB10	FHT(Phospho)ITTSYYR*	Thr3(Phospho)	Mouse/Human	Thr73	Thr73	R* (13C(6)15N(4)	2	1378.6067	689.807
RAB10	FHT(Phospho)ITTSYYR	Thr3(Phospho)	Mouse/Human	Thr73	Thr73	Light	2	1368.5984	684.8028
RAB12	FNS(Phospho)ITSAYYR*	Ser3(Phospho)	Mouse/Human	Ser105	Ser106	R* (13C(6)15N(4)	2	1311.5645	656.2859
RAB29	FT(Phospho)S(Phospho)M(Oxidation)TR*	Thr2(Phospho)Ser3(Phospho)	Mouse/Human	Thr71/Ser72	Thr71/Ser72	R* (13C(6)15N(4)	2	928.2911	464.6492
RAB29	FT(Phospho)SM(Oxidation)TR*	Thr2(Phospho)	Mouse/Human	Thr71	Thr71	R* (13C(6)15N(4)	2	848.3247	424.666
RAB29	FTS(Phospho)M(Oxidation)TR*	Ser3(Phospho)	Mouse/Human	Ser72	Ser72	R* (13C(6)15N(4)	2	848.3247	424.666
RAB35	FRT(Phospho)ITSTYYR*	Thr3(Phospho)	Mouse/Human	Thr72	Thr72	R* (13C(6)15N(4)	2	1397.6489	699.3281
RAB43	FRT(Phospho)ITQSYYR*	Thr3(Phospho)	Mouse/Human	Thr80	Thr82	R* (13C(6)15N(4)	2	1424.6598	712.8335
LRRK2	SNS(Phospho)ISVGEVYR*	Ser3(Phospho)	Mouse	Ser910	Ser910	R* (13C(6)15N(4)	2	1300.5808	650.7941
LRRK2	SNS(Phospho)ISVGEFYR*	Ser3(Phospho)	Human	Ser910	Ser910	R* (13C(6)15N(4)	3	1907.8994	636.638
LRRK2	HSNS(Phospho)LGPVFDHEDLLR*	Ser4(Phospho)	Mouse	Ser935	Ser935	K* (13C(6)15N(2)	2	1925.8781	642.6309
LRRK2	HSNS(Phospho)LGPIFDHEDLLK*	Ser4(Phospho)	Human	Ser935	Ser935	R* (13C(6)15N(4)	2	1348.5808	674.7941
LRRK2	IGDEDGQFPAHR*		Mouse			R* (13C(6)15N(4)	2	1351.6265	676.3169
LRRK2	IGDEDGHFPAHR*		Human			R* (13C(6)15N(4)	3	1360.6268	454.2138
RAB1A	FADDTYTESYISTIGVDFK*		Mouse/Human			K* (13C(6)15N(2)	3	3092.5012	1090.5117
RAB3D	DAADQNFDYMFK*		Mouse/Human			K* (13C(6)15N(2)	2	1472.6242	736.8157
RAB8A	NIEEHASADVEK*		Mouse/Human			K* (13C(6)15N(2)	2	1349.6423	675.3248
RAB8B	NIEEHASSDVER*		Mouse/Human			R* (13C(6)15N(4)	2	1395.6374	698.3224
RAB10	NIDEHANEDVER*		Mouse/Human			R* (13C(6)15N(4)	2	1450.6432	484.2193
RAB10	FHTITTSYYR*		Mouse/Human			R* (13C(6)15N(4)	2	1298.6403	433.5516
RAB10	AFLTLAEDILR*		Mouse/Human			R* (13C(6)15N(4)	2	1271.7233	636.3653
RAB12	DNFNVDEIFLK*		Mouse/Human			K* (13C(6)15N(2)	2	1361.6827	681.345
RAB35	DYDHLFK*		Mouse/Human			K* (13C(6)15N(2)	2	945.4556	473.2314
RAB43	YAGSNIVQLLIGNK*		Mouse/Human			K* (13C(6)15N(2)	2	1497.8515	749.4294

Supplementary Table 2. Detailed demographic and clinical characteristics of blood neutrophil donors.

STUDY ID	Group<br>1=L2_PD<br>2=L2_NMC<br>3=iPD<br>4=C	Mutation<br>1=G2019S<br>2=R1441G	Gender<br>1=Male<br>2=Female	AGE	Disease duration	Age at PD diagnosi	LEDD (mg)	UPDRS III on	UPDRS IV	Site<br>1 Barcelona<br>2 San Sebastian
1	1	1	2	60	17	43	1123	14	4	1
2	2	1	2	37	NA	NA	0	0	0	1
3	2	1	1	69	NA	NA	0	6	0	1
4	2	1	1	53	NA	NA	0	3	0	1
5	4	NA	2	60	NA	NA	NA	NA	NA	1
6	1	2	1	71	1	70	405	25	0	1
7	4	NA	2	67	NA	NA	NA	NA	NA	1
8	2	2	2	63	NA	NA	0	2	0	1
9	2	2	1	60	NA	NA	0	0	0	1
10	1	1	1	77	3	73	750	17	0	1
11	2	1	2	48	NA	NA	0	0	0	1
12	4	NA	1	50	NA	NA	NA	NA	NA	1
13	1	1	2	71	2	68	100	11	0	1
14	4	NA	2	79	NA	NA	NA	NA	NA	1
15	3	NA	1	80	4	75	550	26	1	1
16	1	1	2	47	3	43	705	7	1	1
17	3	NA	1	79	2	77	0	13	0	1
18	3	NA	1	68	3	64	820	12	0	1
19	3	NA	2	56	1	55	315	18	0	1
20	3	NA	2	68	12	55	1010	28	5	1
21	4	NA	2	40	NA	NA	NA	NA	NA	1
22	2	1	1	42	NA	NA	0	0	0	1
23	4	NA	2	68	NA	NA	NA	NA	NA	1
24	3	NA	1	69	8	60	700	26	2	1
25	3	NA	2	74	11	63	1055	13	7	1
26	3	NA	1	78	0	78	450	11	0	1
27	2	1	2	63	NA	NA	0	0	0	1
28	1	1	2	55	1	54	100	11	0	1
29	4	NA	1	57	NA	NA	NA	NA	NA	1
30	3	NA	2	55	5	49	950	12	10	1
31	3	NA	1	74	11	62	838	29	0	1
32	4	NA	2	70	NA	NA	NA	NA	NA	1
33	3	NA	2	68	2	65	300	21	0	1
34	4	NA	1	71	NA	NA	NA	NA	NA	1
35	3	NA	2	66	17	48	510	9	1	1
36	4	NA	1	50	NA	NA	NA	NA	NA	1
37	4	NA	2	82	NA	NA	NA	NA	NA	1
38	4	NA	2	73	NA	NA	NA	NA	NA	1
39	2	1	1	51	NA	NA	0	0	0	1
40	1	1	1	53	20	33	1131	25	8	1
41	4	NA	2	53	NA	NA	NA	NA	NA	1
42	3	NA	1	79	3	75	400	16	0	1
43	1	1	1	80	16	63	400	29	7	1
44	1	1	2	72	10	61	955	10	5	1
45	1	1	2	52	8	43	220	13	5	1
46	2	1	2	48	NA	NA	0	0	0	1
47	3	NA	1	76	3	72	250	10	0	1
48	4	NA	2	73	NA	NA	NA	NA	NA	1
49	2	1	1	45	NA	NA	0	0	0	1
50	4	NA	1	66	NA	NA	NA	NA	NA	1
51	3	NA	2	76	2	73	840	21	0	1
52	1	1	1	66	19	46	400	24	11	1
53	4	NA	2	64	NA	NA	NA	NA	NA	1
54	1	1	2	83	2	81	1164	37	9	1
55	4	NA	2	59	NA	NA	NA	NA	NA	1
56	4	NA	1	64	NA	NA	NA	NA	NA	1
57	3	NA	2	64	14	49	550	34	11	1
58	3	NA	2	78	14	63	750	23	3	1
59	3	NA	1	51	4	46	205	14	0	1
60	2	2	1	62	NA	NA	0	0	0	1
61	4	NA	1	58	NA	NA	NA	NA	NA	1
62	1	1	2	57	4	52	499	10	3	1
63	1	2	1	66	7	58	600	20	0	1
64	4	NA	2	65	NA	NA	NA	NA	NA	1
65	3	NA	1	60	7	52	1100	31	1	1
66	4	NA	2	53	NA	NA	NA	NA	NA	1
67	2	2	1	63	NA	NA	0	0	0	2
68	1	2	2	74	11	63	310	14	0	2
69	1	2	1	62	5	57	300	0	0	2
70	2	2	2	60	NA	NA	0	0	0	2
71	1	2	2	75	9	66	400	4	9	2
72	4	NA	2	45	NA	NA	NA	NA	NA	2
73	1	2	2	86	7	78	550	30	0	2
74	3	NA	1	56	20	37	400	missing	missing	2
75	3	NA	1	75	10	65	900	13	6	2
76	3	NA	1	65	13	52	1865	9	0	2
77	3	NA	1	74	10	64	1617	50	0	2
78	4	NA	2	73	NA	NA	NA	NA	NA	2
79	1	2	2	59	3	56	500	17	0	2
80	4	NA	2	46	NA	NA	NA	NA	NA	2
81	2	2	2	49	NA	NA	0	0	0	2
82	4	NA	2	49	NA	NA	NA	NA	NA	2
83	4	NA	2	55	NA	NA	NA	NA	NA	2
84	4	NA	2	56	NA	NA	NA	NA	NA	2
85	3	NA	2	61	4	57	550	8	0	2
86	4	NA	1	52	NA	NA	NA	NA	NA	2
87	4	NA	2	43	NA	NA	NA	NA	NA	2
88	4	NA	2	64	NA	NA	NA	NA	NA	2
89	3	NA	1	68	5	63	550	23	0	2
90	1	2	1	78	8	70	633	34	0	2
91	1	2	1	63	17	46	1485	42	0	2
92	3	NA	2	53	7	46	825	4	1	2
93	4	NA	2	48	NA	NA	NA	NA	NA	2
94	1	2	2	45	3	42	495	8	0	2
95	1	2	1	74	18	56	1566	43	8	2
96	1	2	2	76	7	69	580	7	0	2
97	1	2	1	61	17	44	974	47	4	2
98	2	2	2	54	NA	NA	0	2	0	2
99	4	NA	1	70	NA	NA	NA	NA	NA	2
100	2	2	2	55	NA	NA	0	1	0	2
101	3	NA	1	61	3	48	1617	34	12	2

Supplementary Table 2. Detailed demographic and clinical characteristics of peripheral blood neutrophil donors. Including group (LRRK2-PD, LRRK2-non-manifesting carrier (NMC), idiopathic PD and control), study and original site ID, specific mutation that participant carriers, age range, disease duration, age at PD diagnosis, *LEDD* is L-dopa equivalent daily dosage, *UPDRS* is Unified Parkinson's Disease Rating Scale[12], part III (motor symptoms) and part IV (motor complications), participant's location site (either Barcelona or San Sebastian). L2_PD = LRRK2-associated Parkinson's disease; L2_NMC = LRRK2 non-manifesting mutation carrier; iPD = idiopathic PD.

Supplementary figure 1. Dose response of the LRRK2 kinase inhibitor MLi-2 in human peripheral blood neutrophils

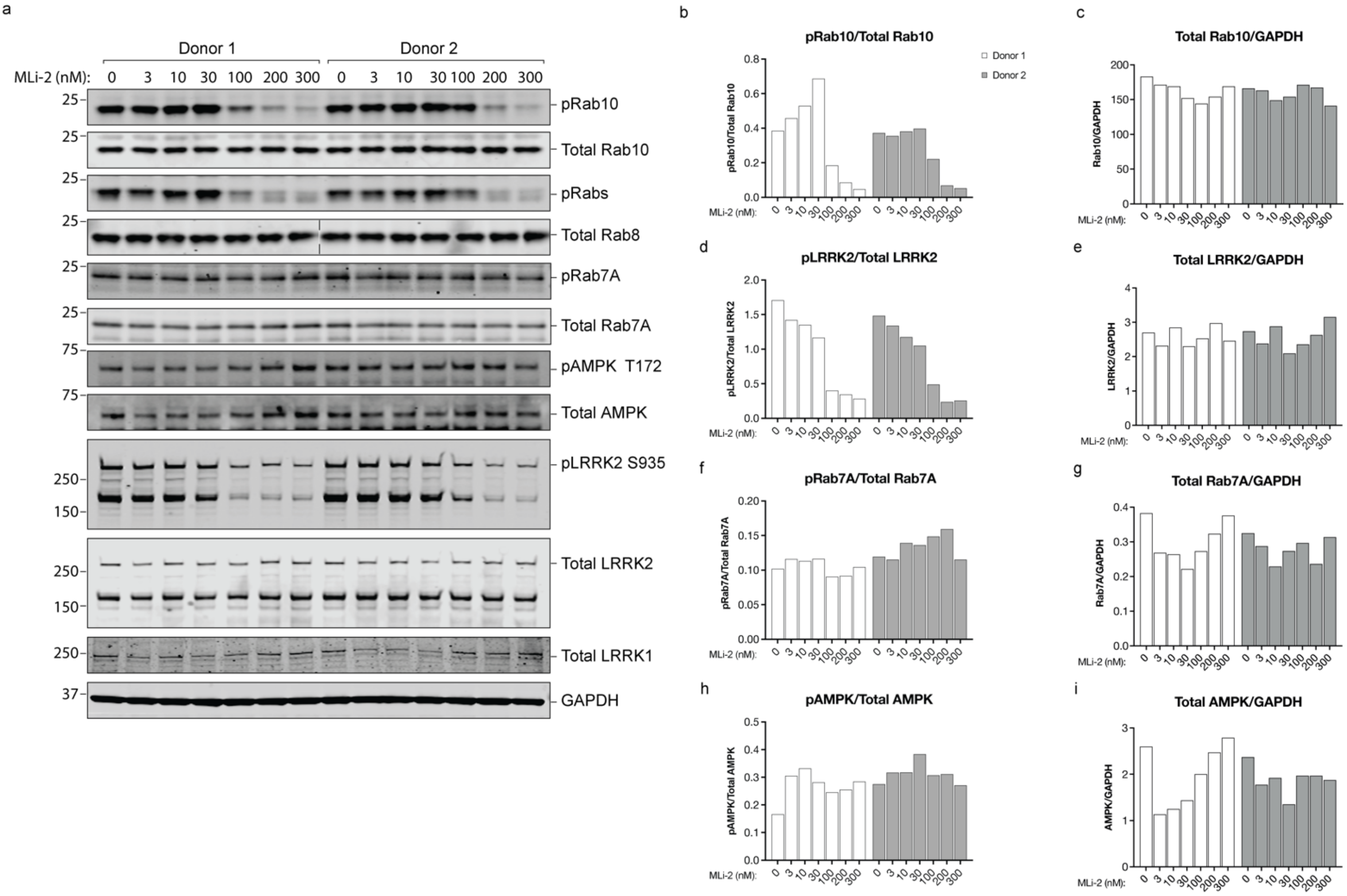

Supplementary figure 1. Dose response of the LRRK2 kinase inhibitor MLi-2 in human peripheral blood neutrophils. (a) Neutrophils were isolated from two healthy donors and treated with the concentrations between 0 and 300nM of MLi-2 as indicated for 30 min. Cells were then lysed and either 10 µg or 20ug (for LRRK1, total and phosphorylated Rab7A) of whole cell extract subjected to quantitative immunoblot analysis with the indicated antibodies and the membranes were developed using the Odyssey CLx scan Western Blot imaging system. (b-i)Quantitation of immunoblots for phospho-Thr73 Rab10/total Rab10 and phospho-Serine 935/total LRRK2 (full length) clearly show significant dephosphorylation of the respective LRRK2 kinase dependent phosphoepitopes at MLi2 concentrations of 30nM up to 300nM (b, d) while total levels of Rab10 and total LRRK2 (full length) don’t significantly change (c, e). In contrast, levels of the LRRK1 dependent Rab7A and AMPK dependent AMPK phosphorylation at Threonine 172 remain largely unchanged (f, h). Total levels of Rab7A (g) and AMPK (i) are also shown.

Supplementary figure 2. Representative Immunoblot analysis of peripheral blood neutrophil samples.

a

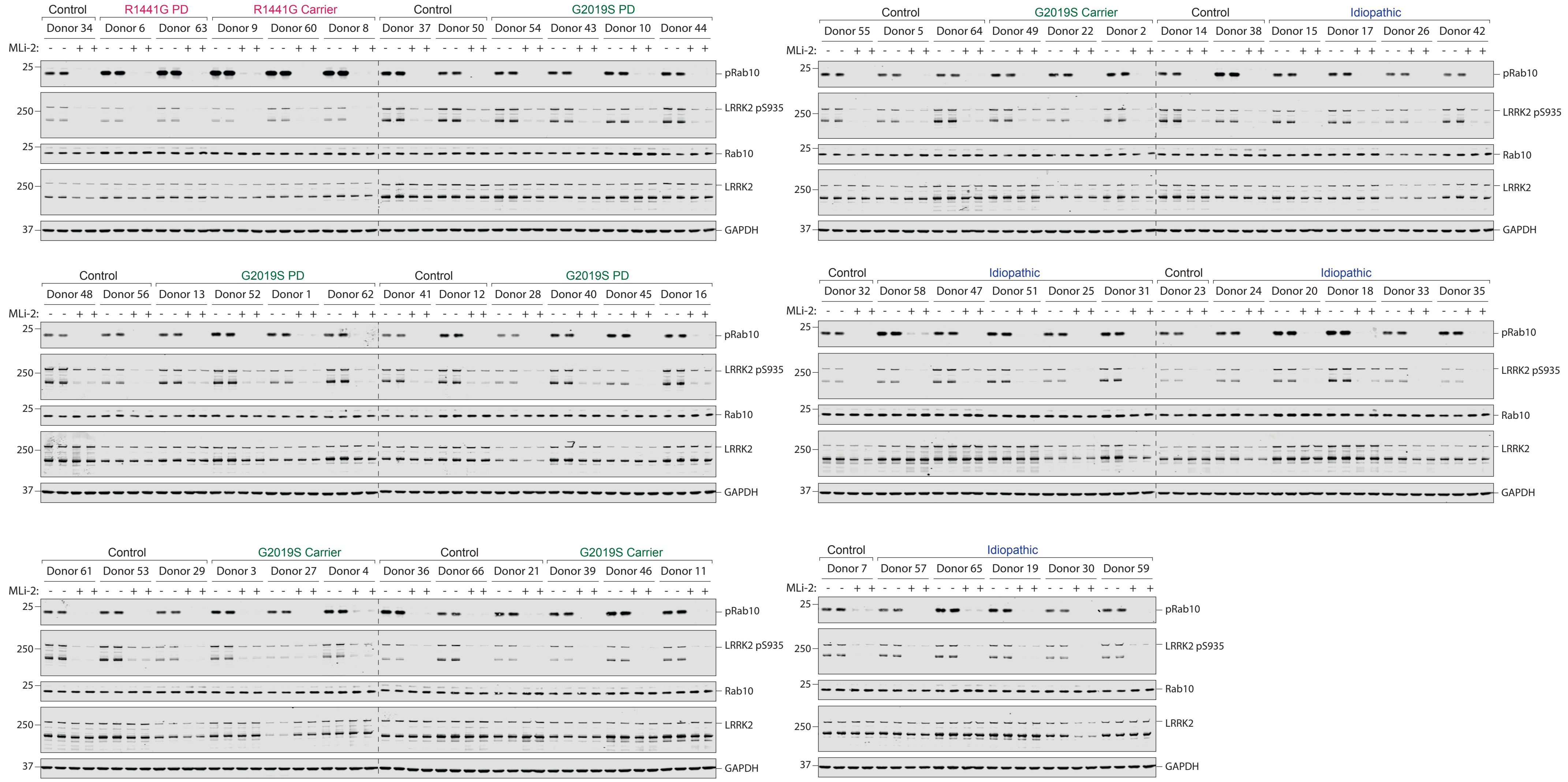

b

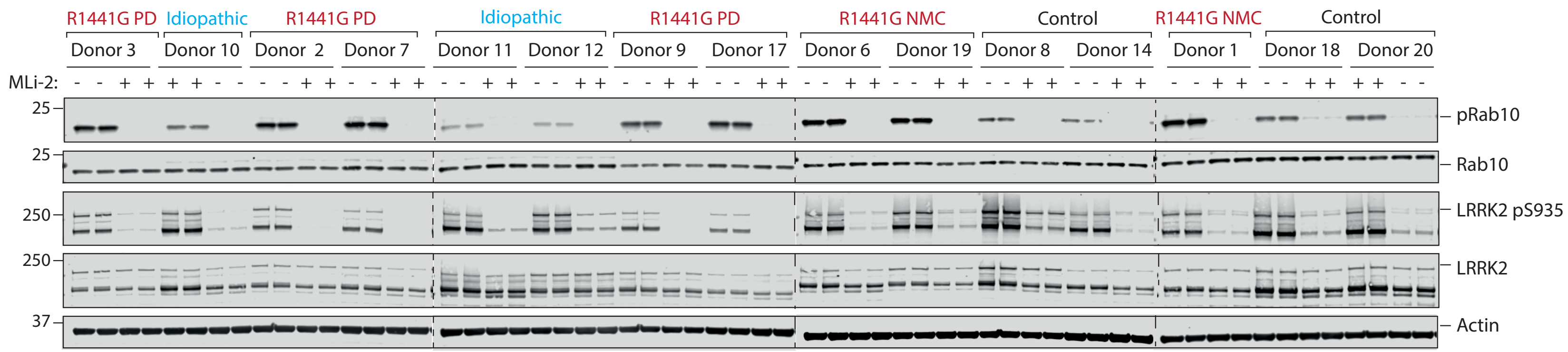

c

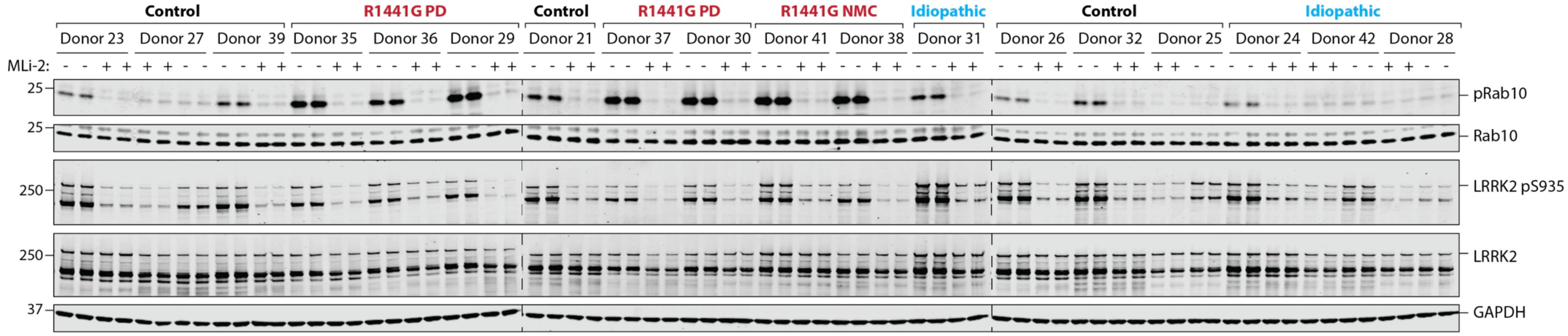

Supplementary figure 2. Representative Immunoblot analysis of peripheral blood neutrophil samples. Neutrophils isolated from fresh peripheral blood were treated with either DMSO vehicle control or the specific LRRK2 kinase inhibitor MLi-2 at a concentration of 200nM for 30 minutes prior to cell lysis. 10 µg of whole cell extracts were then loaded in duplicates and subjected to quantitative immunoblot analysis with the indicated antibodies and the membranes developed using the Odyssey CLx scan Western Blot imaging system. pRab10 and total Rab10 protein as well as Serine 935 and total LRRK2 antibodies were multiplexed and the same internal standard was run on every gel to compare samples run on different gels (not shown). Similar results were obtained in two independent immunoblot experiments of the same extracts. (a) Representative immunoblot for neutrophils derived from the 66 participants from Barcelona (ID 1-66). (b) Representative immunoblot for neutrophils derived from the 35 participants from San Sebastian (ID 67-101).

Supplementary figure 3. Impact of biological sex on LRRK2 dependent Rab10 phosphorylation.

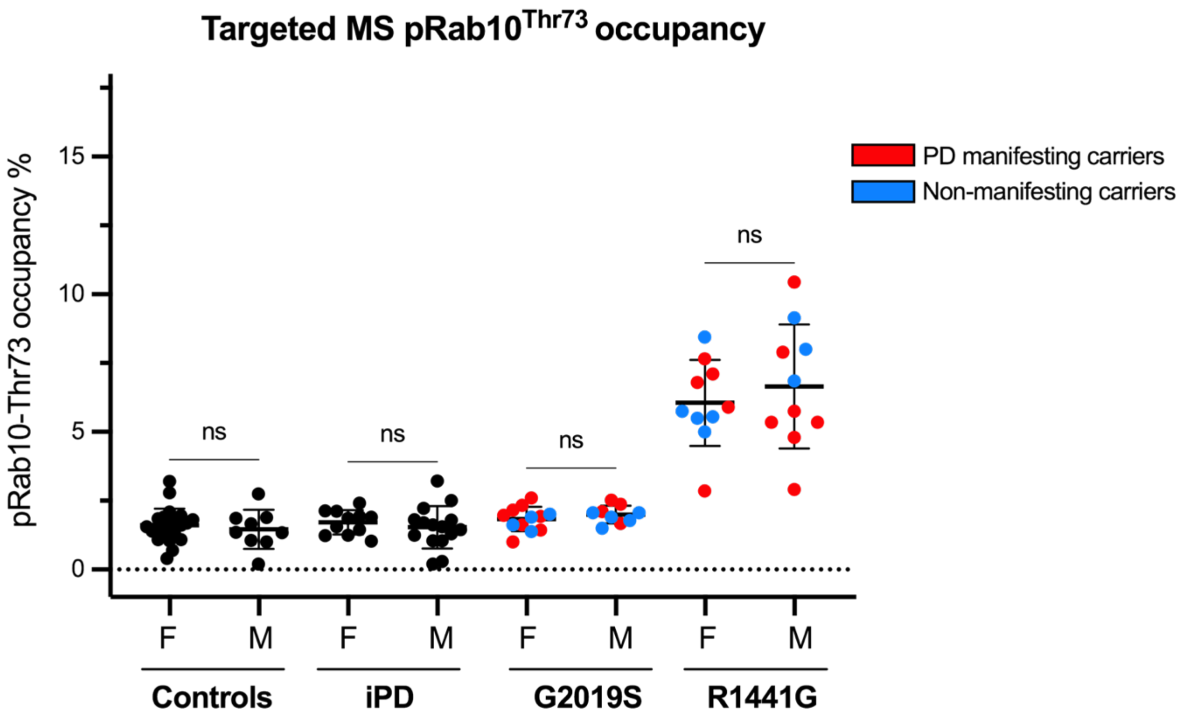

Supplementary figure 3. Impact of biological sex on LRRK2 dependent Rab10 phosphorylation. Quantification of Rab10-pThr73 stoichiometry (%) in DMSO treated neutrophil lysates segregated by gender for each group including controls, iPD and LRRK2 mutation carriers of either the G2019S or R1441G mutation. PD or non-manifesting carriers (NMC) status indicated by colour. One-way ANOVA with multiple comparisons was applied with the mean of females and males for each group being compared with each other. Rab10^{Thr73} phosphorylation occupancy is presented as means ± SD. There was no statistically significant difference between females and males in each group (ns=not significant).

Supplementary table 3: Clinical and pathological characteristics of all brain donors.

Donor	Sample site (1=Columbia, 2=Barcelona)	Clinical diagnosis	Neuropathologic diagnosis	*LRRK2* Mutation status	Sex	Age at death	Cold PMI (hrs:min)	Region for IB analysis (1: Frontal, 2: Occipital)	Thal amyloid phase	NFT Braak stage	CERAD score	NIA "ABC" score	Burden of ADNC	Lewy body Braak stage	LATE-NC stage
**CONTROL GROUP**															
Bar-14	2	Neurologic control	Low ADNC	n.a.	F	81	23:30	1	5	I	moderate	A3B1C2	Low	0	not available
Bar-15	2	Control (Brainstem hemorrhage)	Intermediate ADNC + Brainstem hemorrhage	n.a.	F	90	12:20	1	3	III	moderate	A2B2C2	Intermediate	0	0
Bar-16	2	Neurologic control	PART + Lacunar infarcts	n.a.	M	78	6:00	1	1	I-II	none	A1B1C0	Low	1	0
Bar-17	2	Neurologic control	Low ADNC + AGD stage II	n.a.	F	88	23:59	1	3	II	moderate	A2B1C2	Low	0	0
Bar-18	2	Neurologic control (Metastatic melanoma)	High ADNC + Melanoma metastasis + Lacunar Infarcts	n.a.	F	77	4:30	1	5	V	moderate	A3B3C2	High	0	0
Bar-19	2	Neurologic control	PART + Lacunar infarcts	n.a.	F	83	7:30	1	0	II	none	A0B1C0	Not	0	0
Bar-20	2	Neurologic control	Low ADNC + ARTAG + AGD stage II + LBD + Lacunar infarcts	n.a.	M	86	7:25	1	3	II	sparse	A2B1C1	Low	1	0
Col-7	1	Pneumonia, neurologic control	PART	n.a.	M	87	3:12	1,2	0	III	none	A0B2C0	Not	0	not available
Col-8	1	Past medical history of ischemic heart disease, neurologic control	NDAR	n.a.	M	74	1:33	1,2	0	0	none	A0B0C0	Not	0	not available
Col-9	1	Congestive heart failure and chronic kidney disease, cognitive decline	Low ADNC	n.a.	M	94	07:58*	1,2	2	IV	sparse	A1B2C1	Low	0	not available
**LRRK2-PD GROUP**															
Bar-1	2	PD	PD	c.6055G>A, p.G2019S	M	76	3:30	1	0	I	none	A0B1C0	Not	4	not available
Bar-2	2	PDD	PDD	c.6055G>A, p.G2019S	F	77	8:00	1	0	III	none	A0B2C0	Not	0	not available
Bar-3	2	PD	PD + Intermediate ADNC	c.6055G>A, p.G2019S	F	92	7:00	1	5	IV	frequent	A3B2C3	Intermediate	4	0
Bar-4	2	PD	PD + Low ADNC	c.6055G>A, p.G2019S	F	69	12:30	1	4	II	moderate	A3B1C2	Low	5	0
Bar-5	2	PD	PD	c.4322G>A, p.R1441H	M	69	15:30	1	0	II	none	A0B1C0	Not	0	0
Bar-6	2	PD	PD + PSP +Intermediate ADNC	c.6055G>A, p.G2019S	M	85	17:00	1	4	III	moderate	A3B2C2	Intermediate	0	0
Col-3	1	PD	PD + PSP	c.6055G>A, p.G2019S	M	89	2:05	1,2	0	NA	none	N/A	Not	4	0
Col-5	1	PD	PD + Intermediate ADNC	c.6055G>A, p.G2019S	M	79	07:40*	1,2	3	V	moderate	A2B3C2	Intermediate	6	1
Col-6	1	PD	PD + High ADNC	c.6055G>A, p.G2019S	M	89	12:09	1,2	4	V	moderate	A3B3C2	High	*	1
**NON-LRRK2 PD GROUP**															
Bar-7	2	PD	PD	wt	M	83	14:00	1	0	II	none	A0B1C0	Not	5	0
Bar-8	2	PD	PD + Intermediate ADNC	wt	M	88	16:30	1	3	III	sparse	A2B2C1	Intermediate	4	0
Bar-9	2	PD	PD	wt	F	85	7:00	1	0	IV	none	A0B2C0	Not	5	0
Bar-10	2	PD	PD + Low ADNC	wt	M	83	7:30	1	1	II	sparse	A1B1C1	Low	5	0
Bar-11	2	PDD	PD + Intermediate ADNC	wt	F	82	4:06	1	3	III	frequent	A2B2C3	Intermediate	6	0
Bar-12	2	PD	PD + Low ADNC	wt	M	77	6:25	1	1	III	none	A1B2C0	Low	5	0
Bar-13	2	PD	PD + Low ADNC	wt	M	76	12:00	1	4	II	frequent	A3B1C3	Low	5	0
Col-1	1	PD	PD + PART	wt	F	88	4:00	1,2	0	IV	none	A0B2C0	Not	4	1
Col-2	1	PD	PD + Low ADNC	wt	M	79	10:30*	1,2	3	II	moderate	A2B1C2	Low	6	not available
Col-4	1	PD	PD + Low ADNC	wt	M	81	07:52*	1,2	1	IV	sparse	A1B2C1	Low	4	3

Supplementary table 3: Clinical and pathological characteristics of all brain donors. Demographic data for the postmortem brain samples that were used in this study are shown including the source of the sample (either Columbia University or University of Barcelona), pathologic group including *LRRK2*-associated PD, non-LRRK2 PD, or control, clinical and neuropathologic diagnosis, *LRRK2* mutation status, sex, age at death, cold postmortem interval (PMI) and * frozen PMI where cold PMI was not available, brain region used for immunoblotting, neuropathologic assessment including Thal amyloid phase [7], Braak stage of neurofibrillary changes of the Alzheimer type (neurofibrillary tangles (NFT)) [1], Consortium to Establish a Registry for Alzheimer's Disease (CERAD) score [4] and National Institute on Aging and Alzheimer's Association (NIA-AA) category [3, 5], Braak stage of Lewy body disease [2] as well as Limbic-predominant age-related TDP-43 encephalopathy neuropathologic changes (LATE-NC) score [6] where available. AD=Alzheimer's Disease, ADNC=Alzheimer's disease neuropathologic change, AGD=Argyrophilic grain disease, ARTAG=Aging-related tau astrogliopathy, LBD=Lewy body dementia, NDAR=No diagnostic abnormality recognized, PART=Primary age-related tauopathy, PDD=Parkinson's disease dementia, PSP=Progressive Supranuclear Palsy. * One LB in the claustrum and one LB in cingulate cortex.

Supplementary figure 4: Representative Immunoblot analysis in human brain extracts.

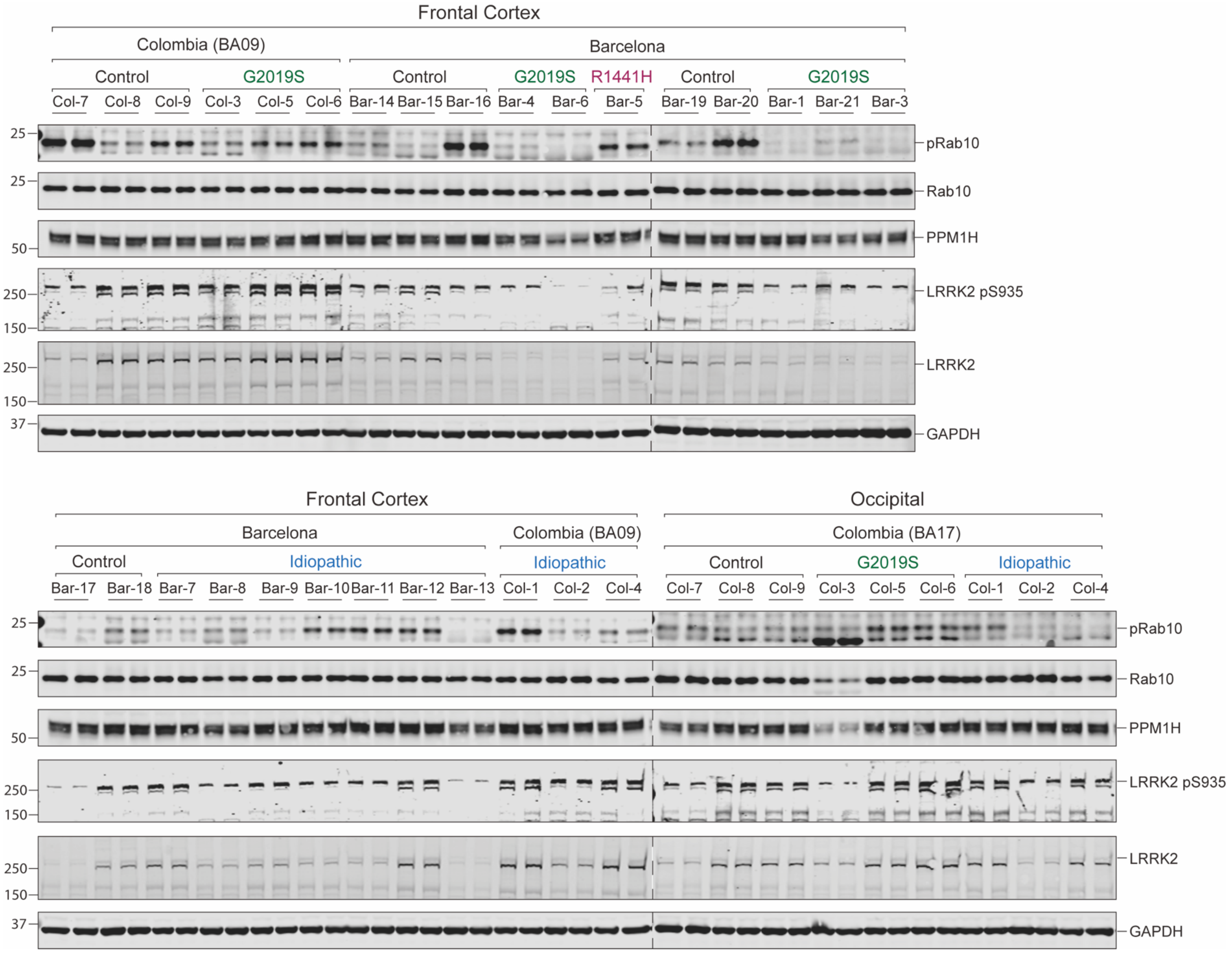

Supplementary figure 4: Representative Immunoblot analysis in human brain extracts. Snap frozen autopsy samples were obtained from matched frontal and occipital cortex samples of 10 individuals including 3 controls, 4 G2019S mutation carriers with PD and 3 iPD from the brain bank at the Columbia University Medical Center in New York, USA and additionally 20 frontal cortex samples from 7 controls, 7 iPD, 5 G2019S and 1 R1441H mutation carriers with PD from the IDIBAPS Biobank at the Hospital Clinic in Barcelona, Spain. Whole tissue lysates from each of these were generated and duplicate loading of 20 µg subjected to immunoblot analysis using the indicated antibodies. pRab10 and total Rab10 protein as well as Serine 935 and total LRRK2 antibodies were multiplexed and the same internal standard was run on every gel to compare samples run on different gels (not shown). Shown here is one of two independent immunoblot experiments used for quantification.

References

Braak H, Alafuzoff I, Arzberger T, Kretzschmar H, Del Tredici K (2006) Staging of Alzheimer disease-associated neurofibrillary pathology using paraffin sections and immunocytochemistry. Acta Neuropathol 112: 389-404 Doi 10.1007/s00401-006-0127-z

Braak H, Del Tredici K, Rub U, de Vos RA, Jansen Steur EN, Braak E (2003) Staging of brain pathology related to sporadic Parkinson's disease. Neurobiol Aging 24: 197-211 Doi 10.1016/s0197-4580(02)00065-9

Hyman BT, Phelps CH, Beach TG, Bigio EH, Cairns NJ, Carrillo MC, Dickson DW, Duyckaerts C, Frosch MP, Masliah Eet al (2012) National Institute on Aging-Alzheimer's Association guidelines for the neuropathologic assessment of Alzheimer's disease. Alzheimers Dement 8: 1-13 Doi 10.1016/j.jalz.2011.10.007

Mirra SS, Heyman A, McKeel D, Sumi SM, Crain BJ, Brownlee LM, Vogel FS, Hughes JP, van Belle G, Berg L (1991) The Consortium to Establish a Registry for Alzheimer's Disease (CERAD). Part II. Standardization of the neuropathologic assessment of Alzheimer's disease. Neurology 41: 479-486 Doi 10.1212/wnl.41.4.479

Montine TJ, Phelps CH, Beach TG, Bigio EH, Cairns NJ, Dickson DW, Duyckaerts C, Frosch MP, Masliah E, Mirra SSet al (2012) National Institute on Aging-Alzheimer's Association guidelines for the neuropathologic assessment of Alzheimer's disease: a practical approach. Acta Neuropathol 123: 1-11 Doi 10.1007/s00401-011-0910-3

Nelson PT, Dickson DW, Trojanowski JQ, Jack CR, Boyle PA, Arfanakis K, Rademakers R, Alafuzoff I, Attems J, Brayne Cet al (2019) Limbic-predominant age-related TDP-43 encephalopathy (LATE): consensus working group report. Brain 142: 1503-1527 Doi 10.1093/brain/awz099

Thal DR, Rub U, Orantes M, Braak H (2002) Phases of A beta-deposition in the human brain and its relevance for the development of AD. Neurology 58: 1791-1800 Doi 10.1212/wnl.58.12.1791
